# Social–Emotional and Educational Needs of Higher Education Students with High Abilities: A Systematic Review

**DOI:** 10.3390/bs15060819

**Published:** 2025-06-14

**Authors:** Marianne Nannings, Marjolijn van Weerdenburg, Petrie J. A. C. van der Zanden, Lianne Hoogeveen

**Affiliations:** Behavioural Science Institute, Radboud University, 6521XZ 4 Nijmegen, The Netherlands; petrie.vanderzanden@ru.nl (P.J.A.C.v.d.Z.); lianne.hoogeveen@ru.nl (L.H.)

**Keywords:** students with high abilities, higher education, social–emotional and educational needs, underachievement

## Abstract

Students with high intellectual abilities in higher education face a significant risk of underachievement due to a range of influencing factors. This systematic review explored their social–emotional and educational needs and examined interventions aimed at addressing both these needs and underachievement. A systematic literature search of a total of 118 records identified 20 social–emotional and 26 educational needs, organized into six overarching categories, illustrating the complex challenges these students face. Of the 42 records investigating an intervention, 38 focused on students directly, three on student advisors and one on teachers. While most interventions addressed multiple student needs, none fully integrated social–emotional and educational aspects. Ultimately, 17 studies examined underachievement, but only 5 implemented an intervention to reduce it. These findings underscore the need for integrated approaches to effectively support higher education students with high abilities.

## 1. Introduction

High intellectual abilities offer immense potential, yet they do not inherently lead to academic success (Almukhambetova & Hernández-Torrano, 2020; Siegle, 2018). Students with high abilities may have distinct social–emotional and educational needs that differ from those of their peers (Bodewes et al., 2021; Schuur et al., 2021; Wiley, 2020). While research has extensively explored these needs in primary education (Hornstra et al., 2018, 2022; VanTassel-Baska & Brown, 2007), in secondary education (Desmet et al., 2020) or across both primary and secondary education (Lo & Feng, 2020; Reis & Renzulli, 2010), there is still a notable shortage of studies specifically addressing higher education. Student success has long been a priority in higher education (e.g., Credé & Niehorster, 2012; Gyurko et al., 2016). Van der Zanden et al. (2019) expanded on this by defining student success as a multi-domain concept, encompassing academic achievement, critical thinking, and social–emotional adjustment. However, research on the academic trajectories of students with high abilities in higher education is sparse (Mendaglio, 2013; Ramos et al., 2022; Schuur et al., 2021; Van den Muijsenberg et al., 2021). Despite their exceptional potential, students with high intellectual abilities are at significant risk of underachievement, often due to factors such as a lack of appropriate challenges, insufficient support, or mismatched educational environments (Almukhambetova & Hernández-Torrano, 2020; Baslanti & McCoach, 2006; Reis & McCoach, 2000; Siegle, 2018). In secondary education, there is a pervasive misconception that these students will naturally excel without additional support (Hately & Townend, 2020; Ritchotte et al., 2014). However, research consistently shows that underachievement is alarmingly common among students who face unique social–emotional and educational challenges that are often overlooked (Al-Khayat et al., 2017; Almukhambetova & Hernández-Torrano, 2020; Bodewes et al., 2021; Schuur et al., 2021; Wiley, 2020). Several studies have examined the dropout rates of students with high abilities, yielding varying conclusions. In 1984 the United States Office of Education estimated that gifted and talented students made up 25 to 30% of school dropouts (Seeley, 1984). More than 20 years later, Matthews (2006) challenged this notion, finding that dropout rates among high school students with high abilities were remarkably low, below 1% across all subgroups.

Little is known about the specific needs of students with high abilities in higher education and effective ways to support their academic success. However, Ramos and Verschueren (2019) did find that Belgian students with high cognitive abilities in higher education, whether at universities or universities of applied sciences, were nearly as likely to experience educational delays as their peers with average cognitive abilities. Although enrichment programs like honors programs have been developed, few studies have investigated whether these programs address the specific needs of students with high abilities (Hinterplattner et al., 2022; Kazemier et al., 2014; Rinn & Plucker, 2019; Wolfensberger, 2015). In the current study, the results of a systematic literature review are presented, focusing on the social–emotional and educational needs of students with high abilities in higher education. By offering a comprehensive synthesis of these specific needs, this review addresses a crucial gap in existing research and contributes valuable insights into how best to support students with high abilities within higher education.

### 1.1. High Ability in Higher Education

Research on the specific needs of higher education students with high abilities presents a unique challenge due to the lack of consensus on the definition of high ability and the diverse terminology used to describe similar concepts, such as “giftedness” and “talent” (Carman, 2013). Vogelaar and Hoogeveen (2020) argued that the term “giftedness” is valuable for recognizing the specific and complex needs of students with (very) high abilities, although they cautioned against using this term as a label, emphasizing its role in understanding rather than categorizing these students. Despite varying definitions, all models of giftedness share the common assumption of exceptional abilities in one or more domains, such as intellectual, artistic, or social areas (Heller, 1991; National Association for Gifted Children, 2013). In scientific literature, high ability is often interpreted through the lens of academic performance (e.g., Desmet et al., 2020; Lo & Feng, 2020; Reis & Renzulli, 2010). Gagné’s Differentiated Model of Giftedness and Talent offers a framework that distinguishes between natural abilities (giftedness) and their transformation into measurable talents, such as academic achievement (Gagné, 2004, 2021, 2023). This transformation is influenced by a combination of intrapersonal factors, such as motivation, volition, and physical or mental capabilities, as well as environmental factors, including access to resources, facilities, and supportive individuals (Gagné, 2004, 2021, 2023; Preckel et al., 2020; Subotnik et al., 2011; Ziegler et al., 2017). The Differentiated Model of Giftedness and Talent (Gagné, 2004, 2021, 2023) highlights the intricate interplay of factors contributing to the achievement and success of students with high abilities, demonstrating that their needs cannot be understood in isolation.

### 1.2. Underachievement

For students in general, reaching their full potential does not occur naturally, and this is equally true for students with high abilities in higher education. In the field of gifted education, there has been a longstanding concern about the potential loss of talent among students with high abilities due to underachievement. Underachievement has been defined in various ways (Reis & McCoach, 2000; Ridgley et al., 2020; Siegle, 2018), but most definitions share a common core: underachievers are students who demonstrate a discrepancy between their expected achievement, based on potential or ability, and their actual performance. Importantly, this discrepancy cannot be attributed to a disability (e.g., a specific learning disability) and must persist over an extended period (Reis & McCoach, 2000; Ridgley et al., 2020). This definition has gained considerable acceptance in contemporary practices for identifying gifted underachievement (Gilar-Corbi et al., 2019; Mofield & Parker Peters, 2019; Steenbergen-Hu et al., 2020) and serves as the foundation for the current study.

### 1.3. Social–Emotional and Educational Needs

Several theories on motivation for learning and academic achievement have elucidated a range of factors associated with underachievement (e.g., Richardson et al., 2012; Robbins et al., 2004; Steenbergen-Hu et al., 2020). Based on the literature, we identified two domains of needs that collectively encompass the factors underlying student requirements, i.e., social–emotional needs and educational needs. Failure to adequately address these needs has been linked to underachievement (e.g., Ridgley et al., 2022; Tan et al., 2023; Van den Muijsenberg et al., 2021).

Social–emotional needs include both intrapersonal and interpersonal factors such as self-esteem, self-regulation, and well-being (Hoogeveen, 2008; Neihart & Yeo, 2017); self-determination (Deci & Ryan, 1994; Ryan & Deci, 2000; Ryan & Moller, 2017); self-efficacy (Bandura, 1989, 1993; Marsh et al., 2016; Zimmerman et al., 2017); and mindset (Dweck & Leggett, 1988; Dweck & Molden, 2017; Dweck & Yeager, 2019). Moreover, regular contact with teachers and peers is recognized as a crucial component of social–emotional development, influencing both interpersonal relationships and intrapersonal growth (Almukhambetova & Hernández-Torrano, 2020; Coenders, 2024; Mambetalina et al., 2023; Van Gerven, 2021; Wissink, 2024).

Educational needs refer to the specific learning supports and instructional strategies necessary for academic success, including appropriate levels of challenge (Scager et al., 2014), differentiated instruction (Tomlinson, 2001), self-regulated learning (Pintrich & De Groot, 1990; Zimmerman, 2002; Zimmerman et al., 2017), and meaningful academic interactions with peers and lecturers (Schmitt & Goebel, 2015). When these underlying needs are met, motivation is naturally enhanced, serving as a key outcome of addressing both domains (Lavrijsen et al., 2021; Ryan & Deci, 2020). Targeted interventions, such as structured programs, strategies, or actions designed to provide appropriate academic, social–emotional, or cognitive support (Reis & Renzulli, 2023), are typically required to address these needs.

### 1.4. Interventions to Reduce Underachievement

Interventions designed for students with high abilities focus on directly addressing their social–emotional and educational needs through strategies such as mentoring (e.g., Lunsford, 2011; Mianehsaz et al., 2022), enrichment programs (e.g., Griffioen et al., 2018; Saleh & Alali, 2022), and personalized learning pathways (e.g., Abdallah, 2024; Mukhamadiyeva & Hernández-Torrano, 2024). These student-centered interventions provide tailored support to help them overcome challenges and optimize their academic potential. In addition to student-focused interventions, interventions aimed at university lecturers and student advisors play a crucial role in creating a supportive learning environment. These interventions focus on increasing educators’ knowledge and experience regarding giftedness, enhancing their ability to recognize and meet the specific needs of students with high abilities. Research has demonstrated that such interventions can significantly impact the learning processes and overall development of students with high abilities in primary and secondary education (Bakx et al., 2019; Hoogeveen, 2008, 2022; Hoogeveen et al., 2005). By improving teachers’ awareness, attitudes, and competencies, these programs help shape the social–emotional and cognitive development of students with high abilities (Hoogeveen et al., 2005). Similarly, interventions targeted at university lecturers and student advisors are expected to yield similar benefits in higher education, fostering an academic environment that promotes both cognitive growth and well-being (Schuur et al., 2021). In the current study, the term student advisor encompasses roles such as student counselors, academic advisors, and other similar support positions.

#### 1.4.1. Interventions Targeting Students

Interventions targeting students with high abilities play a crucial role in addressing their unique needs (Johnson et al., 2022; Pandya, 2023; Sharma et al., 2023). These interventions often focus on enhancing self-regulated learning, fostering a growth mindset, and promoting social–emotional development to support both academic and personal success (Dweck & Molden, 2017; Zimmerman, 2002). By targeting challenges commonly faced by students with high abilities, such as perfectionism and impostor syndrome, these interventions aim to mitigate barriers that can hinder achievement and well-being (Lee et al., 2021; Neumeister, 2004; Rice et al., 2006; Ridgley et al., 2022). One of the theoretical concepts underlying many of these interventions is personalized learning, which emphasizes the tailoring of instruction, support, and pacing to meet individual student needs, preferences, and strengths. Walkington and Bernacki (2020) underscore the importance of personalized learning as a broadly applicable educational approach that benefits all students by promoting engagement, autonomy, and deeper learning. This approach is also relevant for students with high abilities, whose academic profiles often diverge from standardized curricula and who may especially benefit from adaptive, learner-centered approaches. Another theoretical concept is Jigsaw-based cooperative learning, in which each student becomes an “expert” on a specific part of the material and then teaches it to their peers, provide a useful framework for structuring peer-to-peer teaching in ways to promote shared responsibility, engagement, and deeper understanding among learners (Ruiz-Gallardo & Reavey, 2019). Additionally, specialized coaching programs and peer support initiatives have demonstrated effectiveness in helping students with high abilities navigate the academic and social complexities of higher education (M. C. Miller, 2017; Schuur et al., 2021). For instance, mentoring and buddy interventions specifically designed to foster peer-to-peer relationships, particularly for first-year students, have been beneficial in easing transitions and enhancing social integration (Fewster-Young & Corcoran, 2023). Furthermore, some approaches offer promising avenues for fostering collaborative learning and maximizing the potential of students with high abilities, such as peer-to-peer teaching (with students teaching and learning from one another) and ability tracking, which involves grouping students according to their skill levels to provide tailored instruction (Kimbrough et al., 2022). The goal of these interventions is to address academic challenges, to foster meaningful peer connections, to strengthen resilience, and to ensure that students with high abilities can thrive in higher education settings.

#### 1.4.2. Interventions Targeting Teachers and Student Advisors

Teachers’ attitudes toward giftedness play a pivotal role in shaping their interactions with students with high abilities, directly influencing these students’ academic achievement and overall well-being. Such attitudes can range from supportive perspectives to (often unconscious) negative stereotypes, such as perceiving students with high abilities as socially or emotionally deficient (Baudson & Preckel, 2013; Hoogeveen et al., 2005; Geake & Gross, 2008; Mattheis, 2018; Neihart & Yeo, 2017). Cross-cultural studies further reveal significant variations in teachers’ attitudes toward giftedness, highlighting the influence of contextual and cultural factors (Akgül, 2021). These findings underscore the critical need for increased awareness and professional development among educators to address the unique challenges faced by students with high abilities.

Research so far has emphasized the need for specialized training programs aimed at equipping both educators and student advisors with the skills necessary to support students with high abilities effectively. For example, Guasch et al. (2010) and Oonk et al. (2020) advocate for professional development initiatives tailored to the unique characteristics of students with high abilities, while Van den Muijsenberg et al. (2021) highlight the value of coaching programs specifically designed for students with high abilities in higher education. Such efforts not only aim to address the academic needs of these students but also recognize the importance of fostering their personal growth and well-being. The importance of targeted interventions is further highlighted in broader research on giftedness in higher education. Studies demonstrate that mentoring, advising, and personalized support systems are critical in aligning educational approaches with the needs and interests of students with high abilities (Abeysekera, 2014; Al-Khayat et al., 2017; Almukhambetova & Hernández-Torrano, 2020; M. C. Miller, 2017; Schuur et al., 2021; Siegle et al., 2014; Sierra et al., 2015). These interventions work to enhance motivation, which in turn helps reduce the risk of academic underachievement (García-Martínez et al., 2021). Despite the progress made, gaps remain in addressing the professionalization of student advisors. While the potential benefits of advisors who understand the characteristics and needs of students with high abilities are evident, such as more effective guidance and the prevention of underachievement, empirical research in this area is still limited. This underscores the need for future studies to explore how advisor training and expertise can contribute to improved outcomes for students with high abilities in higher education.

### 1.5. Current Study

The objectives of the current study are to deepen the understanding of the specific social–emotional and educational needs of students with high abilities in higher education as identified in the literature, and to systematically review the effectiveness of interventions addressing these needs and reducing underachievement. The current study examined the specific needs identified in the literature, the types of interventions developed to reduce underachievement, the needs that were addressed by these interventions, and the extent to which these reduce underachievement. By addressing these objectives, this research aims to bridge a significant gap in higher education literature and support the development of evidence-informed practices that empower students with high abilities to reach their full potential. In line with the current study’s focus on pedagogical practices and teacher–student interaction, the term “teacher” is used throughout this manuscript to refer specifically to individuals engaged in teaching in higher education settings. The following research questions were formulated:

1. Which specific social–emotional needs and educational needs of students with high abilities in higher education are identified in research?
2. What types of interventions have been developed to reduce underachievement among students with high abilities in higher education?
3. Which needs are addressed by these interventions?
4. To what extent do these interventions impact the reduction of underachievement?

## 2. Method

The current study followed a systematic review approach, which is a structured and comprehensive method for identifying, evaluating, and synthesizing research evidence to answer specific research questions (Petticrew & Roberts, 2006). The study was pre-registered in the OSF registries on 12 March 2024 (blinded for review). It has been conducted in accordance with the pre-defined methodology and research framework as outlined in the pre-registration. The only modification to the original plan is the extension of the end date from September 2024 to April 2025, along with the removal of the initially proposed second research question, which was determined to be no longer pertinent to the revised scope of the study. Adhering to PRISMA guidelines (Moher et al., 2010), the study followed a structured and transparent methodology to ensure a rigorous and systematic synthesis of evidence.

### 2.1. Search Strategy

Systematic searches were conducted in ERIC, PsycINFO, Scopus, and Web of Science, chosen for their comprehensive coverage of academic research. The search, first conducted on 28 September 2023, and updated on 22 February 2024, was restricted to English-language, peer-reviewed articles published between 1 January 2000 and 22 February 2024. The review focused on literature from 2000 onward to ensure alignment with contemporary higher education practices and evolving educational paradigms. This period also marks a shift in giftedness research, with increasing attention to underachievement, social–emotional needs, and contextual influences on high-ability students, making post-2000 literature particularly relevant to the scope of this review. The search terms combined variations of “high ability,” “higher education,” and “achievement” or “social-emotional well-being.” The search strategy was peer-reviewed by two information specialists to ensure its rigor. The search terms used included the following: “(gifted* or high ability or highly able or high achiev* or high iq or high* intelligen* or talented or accelerated student* or academic accelerat* or advanced student* or exceptional student* or excellent student* or genius* or academic abilit* or creative intelligen* or intellectual* overexcitab*) AND (higher education or postsecondary or post secondary or tertiar* education or universit* or colleg* or polytechnic* or undergraduate* or under graduate* or firstyear student* or first year student* or freshmen or freshman or freshwomen or freshwoman or bachelor student* or sophomore*) AND (underachiev* or underperform* or poor perform* or low perform* or failing grade* or failure* or underproduc* or expect* or result* or achiev* or perform* or success* or attain* or credit* or grade-point average* or adjustment or motivat* or well-being or wellbeing or learn* or difficult* or experience* or need* or barrier* or obstacle* or demand* or social-emotional or socio-emotional or socioemotional or at risk or challeng*)”.

**Figure 1:**
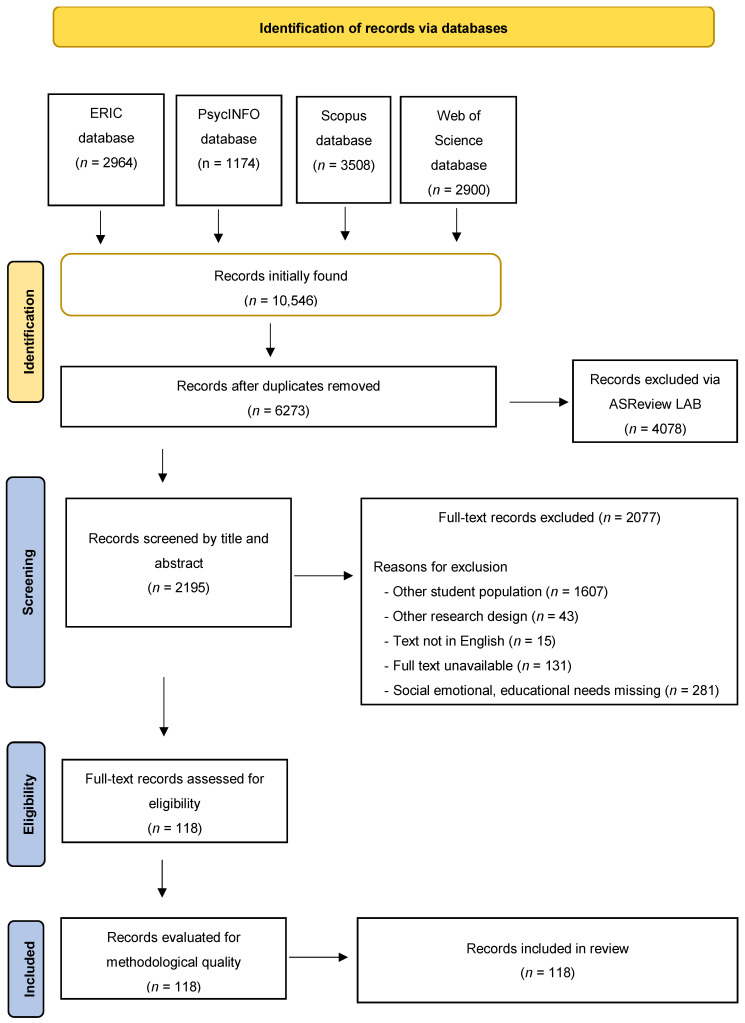
Flow diagram of search and selection process.

To identify relevant studies, we applied pre-defined inclusion and exclusion criteria focusing on quantitative or mixed methods (i.e., quantitative and qualitative) research that examined social–emotional or educational needs and achievement-related interventions among higher education students with high abilities. Studies involving primary, secondary, or twice-exceptional students were excluded to maintain a clear focus on the target population. Figure 1 presents a flow diagram of the search and selections process. Initially, a total of 6273 records were found in various databases after duplicates had been removed. Title and abstract screening using ASReview LAB reduced the pool to 2195 records. Full-text articles were then assessed for eligibility, leading to the exclusion of 2077 articles for reasons such as unsuitable research designs, non-English texts, unavailability of full texts, or insufficient focus on the targeted needs. Ultimately, 118 records met the inclusion criteria and were incorporated into the final review. All records were organized using EndNote, and systematic data analyses were conducted in Excel to extract study characteristics, population details, interventions, and outcomes.

The quality of the included records was assessed using elements of the Mixed Methods Appraisal Tool (MMAT) (Nha Hong et al., 2018), which evaluates factors such as sampling validity, measurement reliability, and methodological integration in mixed methods designs. Of the reviewed studies, 64 (54%) were classified as good, 36 (31%) as sufficient, and 18 (15%) raised quality questions. These questions primarily stemmed from the absence of reported reliability indices, limiting a full assessment of the instruments’ internal consistency and construct validity, as well as issues such as small sample sizes and the inclusion of students from only one course or a single institution, which raises questions about the generalizability of the findings. A detailed overview of the quality assessment is available on request.

### 2.2. Analysis

A narrative synthesis was conducted to integrate findings related to the research questions, following Petticrew and Roberts’ (2006) approach of systematically summarizing and analyzing included studies through thematic analysis, data tabulation, and relationship exploration. To answer the first research question, an overview was created in Excel, selecting criteria such as study design, population, intervention type, and main findings. This structured approach facilitated a systematic comparison of studies and enabled the identification of thematic categories with themes categorized by their prevalence in the literature. Subsequently, to address the second research question, an analysis was conducted on the interventions described in the included literature. Studies were categorized based on the type of intervention (e.g., mentoring programs, curriculum differentiation, social–emotional support initiatives, and/or coaching), their implementation contexts, and their target outcomes. This categorization provided insight into the range of approaches used to support students with high abilities and reduce underachievement. For the third research question, a comparison was made between the identified interventions and the social–emotional and educational needs reported in the literature. This analysis focused on determining which specific needs were explicitly targeted by each intervention. Finally, to answer the fourth research question, the selected studies were further analyzed to assess the reported outcomes and effectiveness of interventions in reducing underachievement.

## 3. Results

### 3.1. Identified Needs of Higher Education Students with High Abilities

The systematic review of the 118 records identified a total of 46 distinct needs, which were classified into two domains: 20 social–emotional needs and 26 educational needs. Table 1 presents an overview of the reviewed studies, including the authors’ names, year of publication, country where the study was conducted, research design, sample size, instruments, and key findings. As can be seen in Table 1, the needs were measured with a variety of instruments, mostly questionnaires, secondary data analysis, test administration, and qualitative instruments such as interviews and observations.

An overview of all needs and frequencies that were found in the literature is presented in Appendix A for social–emotional needs and in Appendix B for educational needs. To ensure a structured and comprehensive analysis, these needs were grouped into overarching categories based on thematic similarities. The analyses identified three distinct categories within each domain, providing a clear framework for understanding the specific challenges and requirements associated with each area.

The three most prominent needs within each respective category are discussed below. The sources referenced in the subsequent discussion were selected as examples of studies of sufficient to good quality in which the particular need was given a prominent focus in the research. The ranking reflects the relative importance of each category based on its prevalence in the literature, ensuring that the most pressing areas of need are addressed with priority.

### 3.2. Identified Social–Emotional Needs of Students with High Abilities

The domain of social–emotional needs highlights key factors that influence the well-being and development of students with high abilities. These needs were categorized into three categories: (1) Self-Perception, Motivation, and Performance Expectations, which all three explore the way students view themselves and navigate external demands; (2) Psychological Well-Being and Emotional Regulation, which focus on managing stress and maintaining mental health; and (3) Social Integration and Acceptance, which examine students’ relationships and sense of belonging.

#### 3.2.1. Self-Perception, Motivation, and Performance Expectations

**Figure 2:**
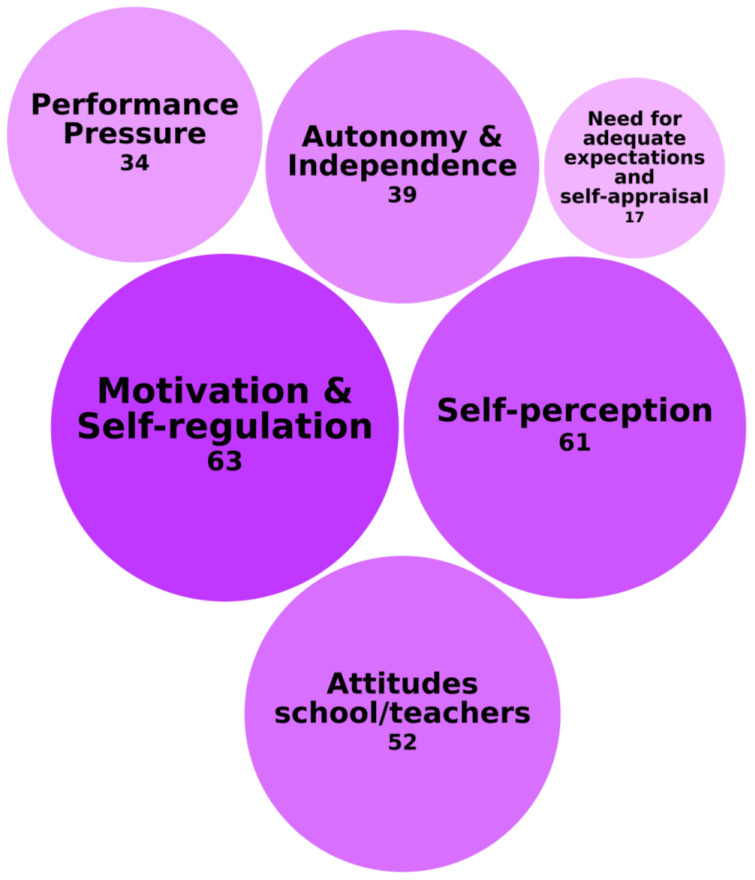
Self-Perception, Motivation, and Performance Expectations: specific needs within this category and their frequency in 118 records.

The most frequently investigated category of social–emotional needs among students with high abilities is called Self-Perception, Motivation, and Performance Expectations. This category concerned the way students view themselves, manage motivation and self-direction, and handle external expectations. Figure 2 illustrates this category of needs, the specific needs encompassed within it, and the frequency with which these needs were identified across the 118 records.

Motivation and self-regulation, which are closely intertwined, were identified in 63 of 118 records as a crucial factor in academic persistence and success. Al-Shabatat et al. (2010) identified self-confidence, perseverance, and ambition as key motivational drivers that help students stay engaged and overcome academic challenges. Their findings illustrate how intrinsic motivation supports long-term academic persistence, making it a critical factor in preventing disengagement. This aligns with Tsai and Fu (2016), who linked low motivation to underachievement, emphasizing the need for deeper learning strategies to sustain academic performance. Similarly, O’Conner (2003) found that supportive academic environments enhance motivation. Together, these studies highlight the importance of both internal and external motivational influences, reinforcing the need for targeted interventions to foster self-belief, goal setting, and engagement in students with high abilities.

In 61 of 118 records, the need for a realistic and positive self-perception was identified as a significant factor in academic success. By shaping students’ confidence and self-efficacy, self-perception influences academic persistence, engagement, and overall performance. This underscores the importance of fostering self-awareness and resilience in students with high abilities to support their long-term achievement. For example, Almukhambetova and Hernández-Torrano (2020) found that students with high abilities often struggle academically when surrounded by equally high-achieving peers, which can negatively affect their self-perception. This comparative self-assessment often leads to doubts about their own abilities and a reluctance to seek help, further reinforcing feelings of inadequacy. Similarly, Kerr and Kurpius (2004) demonstrated that interventions aimed at self-esteem development improve persistence in math and science, suggesting that bolstering self-perception can enhance academic resilience. Maranges et al. (2023) further linked brilliance beliefs to academic choices, showing that women who perceived themselves as less inherently brilliant were more likely to opt for fields perceived as requiring lower levels of innate talent, illustrating how self-perception directly shapes educational and career trajectories.

In addition, a considerable number of studies indicated that attitudes toward school, found in 52 of 118 records, influence student engagement and performance, shaping motivation and academic persistence. For example, Baslanti and McCoach (2006) revealed that underachievers exhibited fewer positive attitudes toward school and teachers, affecting motivation. Mahenthiran and Rouse (2000) demonstrated that students who worked with a friend showed higher engagement and improved academic performance, highlighting the role of social support in fostering positive attitudes toward school. Tindage et al. (2024) explored this in the context of students of color, showing how positive master narratives acted as microaffirmations, whereas negative narratives contributed to academic self-doubt.

#### 3.2.2. Psychological Well-Being and Emotional Regulation

**Figure 3:**
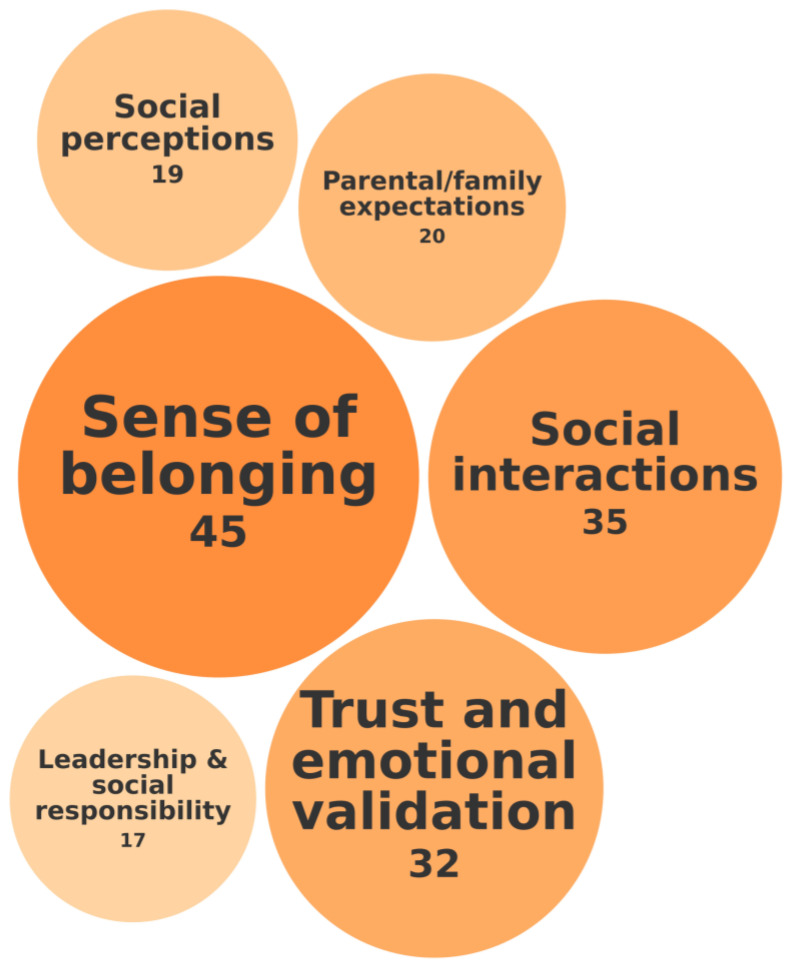
Psychological Well-Being and Emotional Regulation Needs: specific needs within this category and their frequency in 118 records.

The second category was called Psychological Well-Being and Emotional Regulation. It encompasses sense of belonging, social interaction, stress management, and identity development. This category concerns the way in which students navigate stress, perfectionism, self-awareness, and identity formation. Figure 3 depicts this category of needs, the specific needs it includes, and the frequency with which they were identified in the 118 records.

Findings from the systematic review showed that sense of belonging, investigated in 45 of 118 records, is a fundamental need. It is defined as the extent to which a student feels accepted, valued, and connected within their social and academic environment, and reflects the perception of being an integral member of a community, whether among peers, faculty, or within the institution. This need plays a critical role in emotional well-being, social integration, academic adjustment, and academic success.

For example, Almukhambetova and Hernández-Torrano (2020) underscored that a strong sense of belonging is crucial for the successful transition of students with high abilities to university life. Their findings show that students who feel connected to their institution, often facilitated by on-campus living and active social engagement, report higher levels of academic success, social integration, and overall well-being. In contrast, a diminished sense of belonging is linked to social isolation, increased emotional distress, and greater difficulty adapting to academic life. Similarly, Bauer et al. (2023) found that biased talent self-concepts, especially among first-generation students who perceive themselves as less inherently talented, contribute to a diminished sense of belonging in environments that place a high value on innate ability. When students internalize the belief that they lack the “natural” talent prized by their academic settings, they are less likely to feel fully integrated and accepted, which negatively affects their academic engagement and overall adjustment. Further highlighting the importance of sense of belonging, Domínguez-Soto et al. (2023) examined its role in reducing impostor feelings among students. Their study found that first-year students reported higher impostor ratings, which decreased over time as they built achievements or, in some cases, dropped out. Based on their findings, the authors recommend fostering a sense of belonging as a key strategy to help students overcome impostor syndrome and enhance retention and academic success.

Furthermore, 35 of 118 records reported that social interaction is essential for student success. For example, Almukhambetova and Hernandez-Torrano (2021) found that even well-adjusted students struggle with social integration, highlighting that strong academic performance alone does not guarantee social adaptation. Tushnova (2020) argued that effective social interaction is a vital need for mathematically gifted students, whose specialized social–perceptual abilities, such as empathy, emotional intelligence, and the recognition of nonverbal cues, play a crucial role in navigating interpersonal relationships and adapting socially in academic environments. Similarly, Rice et al. (2006) highlighted that social connectedness is critical for high-achieving students, as strong social interactions help buffer the negative effects of maladaptive perfectionism, reducing stress and depression while enhancing academic integration. In essence, supportive peer and faculty relationships are indispensable for the emotional well-being and academic success of students with high abilities.

Finally, in 31 of 118 records, trust and emotional validation were found to play a critical role in reducing anxiety and fostering inclusion among students with high abilities. For example, Palmiero et al. (2023) linked cognitive flexibility to trust in diverse social settings, while Lee et al. (2021) found that perfectionism and imposter syndrome intensified students’ need for validation. Liu (2014) demonstrated how structured learning environments reduced anxiety by creating a supportive academic atmosphere.

#### 3.2.3. Social Integration and Acceptance

**Figure 4:**
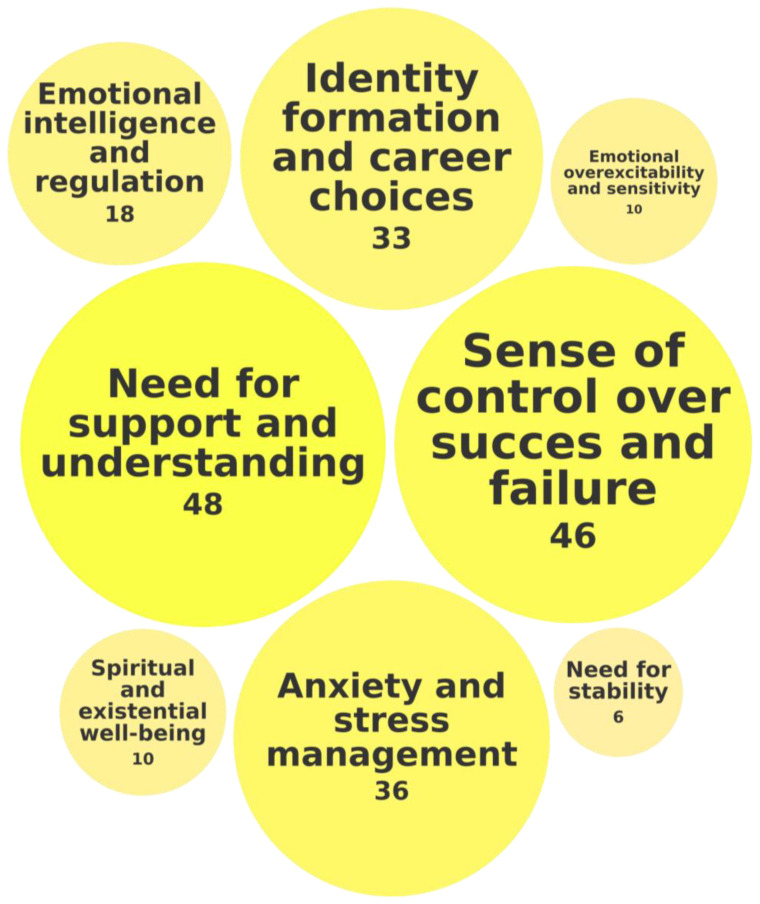
Social Integration and Acceptance: specific needs within this category and their frequency in 118 records.

The third category of identified social–emotional needs was called Social Integration and Acceptance and concerned the way in which students with high abilities navigate social settings, build relationships with peers and adults, and respond to social expectations that influence their emotional well-being. Figure 4 presents an overview of this category of needs, detailing the specific needs it comprises and the frequency of their identification across the 118 records.

Notably, 48 of 118 records emphasized that social and spiritual support are essential for emotional validation, helping students feel understood and accepted. For example, O’Conner (2003) found that students sought academic support from lecturers and peers, and the personal tutor system was especially valuable in the first year, with some students receiving extra assignments and study advice. Similarly, Van den Muijsenberg et al. (2021) stressed the importance of enhanced social and academic integration, while Yusmansyah et al. (2019) found that religious support can help students overcome internal barriers to academic success.

In 46 of 118 records, the need for a sense of control over success and failure is identified as a crucial aspect of Social Integration and Acceptance. When students with high abilities believe they can influence outcomes through effort, they feel more competent, confident, and connected. This perceived control, linked to self-efficacy, reinforces self-esteem and belonging, while its absence can lead to helplessness and isolation. For example, Smith (2006) found that early academic setbacks increased college dropout risks, shaping control perceptions. Snyder et al. (2014) showed that students who viewed giftedness as fixed engaged in self-handicapping after failure. Strayhorn (2009) found that 88% of high-achieving Black collegians felt pressure to prove their intellect despite prior success, illustrating how societal expectations can erode perceived control, leading to stress, self-doubt, and disengagement.

In 36 of 118 records, the need to manage anxiety and stress underscores the challenges of balancing academic pressures and expectations. For example, Rice et al. (2006) found that both adaptive and maladaptive perfectionism were strongly linked to perceived stress, depression, and academic adjustment. Li et al. (2023) identified three stress profiles among freshmen, revealing that perceived stress significantly predicted emotional distress, and that resilience alone was not sufficient to mitigate stress effects, highlighting the critical need for targeted stress management strategies.

### 3.3. Identified Educational Needs of Students with High Abilities

The domain of educational needs addressed key factors that support students with high abilities in maximizing their potential. These needs are categorized into three categories, i.e., (1) Curriculum Design and Instructional Strategies; (2) Academic and Social Support Systems; and (3) Teacher Training and Pedagogical Expertise.

#### 3.3.1. Curriculum Design and Instructional Strategies

**Figure 5:**
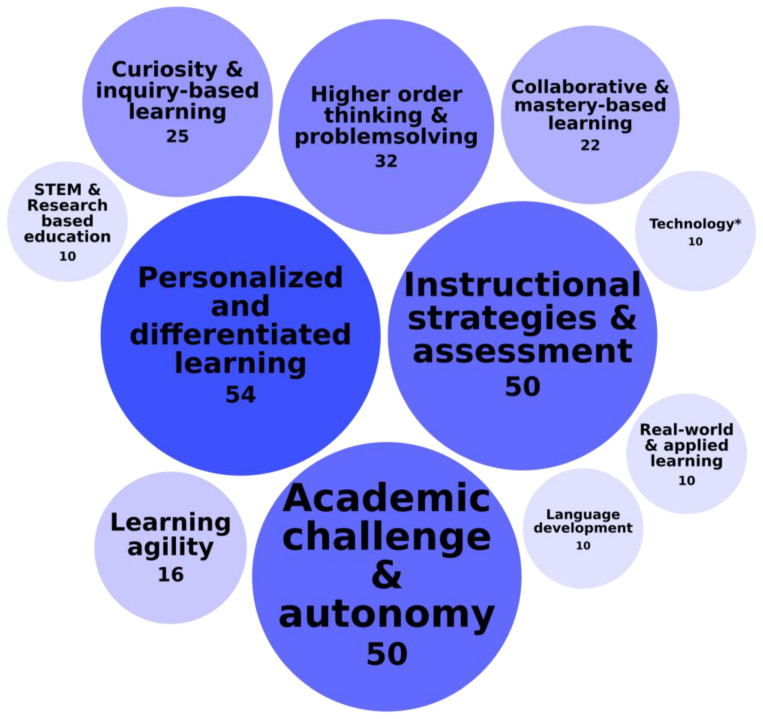
Curriculum Design and Instructional Strategies: specific needs within this category and their frequency in 118 records. Note: * indicates technology-enhanced learning.

The first category of identified educational needs was called Curriculum Design and Instructional Strategies. This category emphasized the importance of intellectually stimulating and flexible learning experiences that foster critical thinking, problem-solving, and academic engagement for students with high abilities. Figure 5 presents an overview of this category of needs, detailing the specific needs it comprises and the frequency of their identification across the 118 records.

Within this category called Curriculum Design and Instructional Strategies, personalized and differentiated learning was the most frequently investigated need, appearing in 54 of 118 records, emphasizing the importance of tailoring instruction to individual strengths, interests, and learning paces. For example, Noble and Childers (2008) identified a fundamental need for intellectual challenge, academic engagement, and appropriate pacing, showing that students with high abilities thrive in environments that allow them to progress at a pace suited to their abilities. This reinforces the necessity of a flexible curriculum design to prevent disengagement and underachievement. Similarly, Griffioen et al. (2018) highlighted the effectiveness of differentiated instruction, while Woody and Parker (2012) demonstrated how personalized learning fosters self-directed learning strategies among high-achieving non-music students. Their study showed that this approach enables students to develop autonomous learning methods and actively seek peer support in a performance project.

The second most frequently identified educational need concerned instructional strategies and assessment, investigated in 50 of 118 records, emphasizing the importance of teaching methods that ensure appropriate challenge, engagement, and academic growth to support students with high abilities. For example, Erdogan (2017) identified a critical need for pre-service teacher training in gifted education, finding that many instructors lacked the necessary preparation to effectively engage and challenge gifted students. Similarly, Derado et al. (2016) found that the Point Reward System helped prevent underperformance by providing continuous reinforcement, reducing the pressure of high stakes testing, and fostering sustained engagement. Unlike traditional assessments, which relied on a few high-impact exams, a Point Reward System rewards incremental progress and effort, encouraging students to stay committed to their learning. By making academic success feel more attainable and reinforcing positive learning behaviors, this system lowered dropout rates and created a more supportive and engaging learning environment.

The third most frequently mentioned educational need concerned academic challenge and autonomy, investigated in 50 of 118 records. This need refers to the necessity of providing intellectually stimulating tasks and opportunities for independent learning. Unlike teaching methods and tailored instruction, which focus on how content is delivered and adapted to student needs, academic challenge and autonomy emphasize what students learn and the level of control they have over their learning process. It highlights the importance of allowing students with high abilities to explore complex ideas, take ownership of their learning, and work at a pace that matches their capabilities to maintain engagement and prevent underachievement. Griffioen et al. (2018), for instance, found that cognitively able students in vocational higher education often lacked sufficient intellectual stimulation, which negatively impacted their academic satisfaction and engagement. Sa Ngiamsunthorn (2020) highlighted challenge-based learning, problem-solving, and project-based learning as key strategies to enhance engagement. Gojkov et al. (2015) emphasized the importance of autonomy, showing that despite strong intellectual functioning, gifted master’s students lacked opportunities for independent learning, problem-solving, and critical thinking.

#### 3.3.2. Academic and Social Support Systems

**Figure 6:**
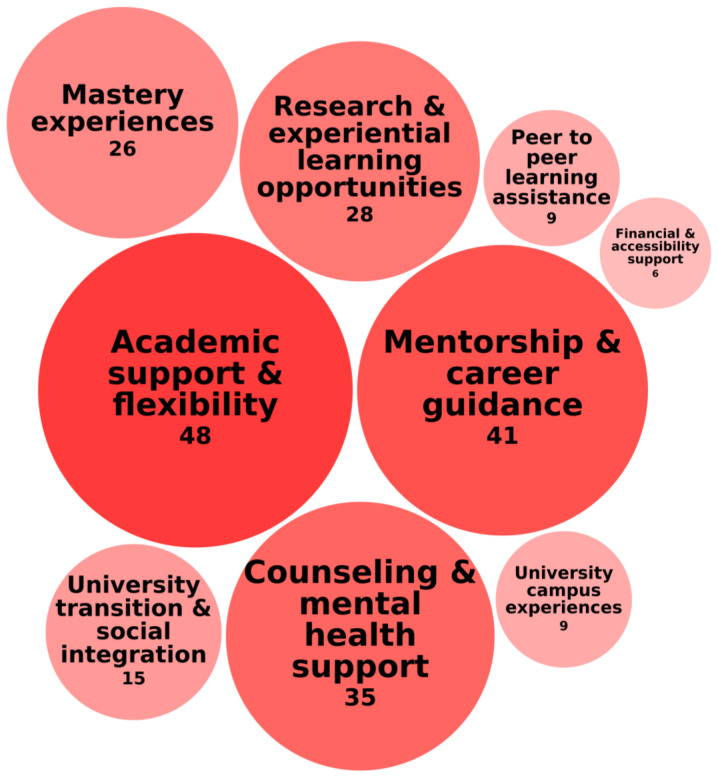
Academic and Social Support Systems: specific needs within this category and their frequency in 118 records.

The second category of educational needs was Academic and Social Support Systems, which highlight the importance of structured academic and emotional support to help students with high abilities navigate their educational and career paths. Figure 6 illustrates this category of needs, the specific needs encompassed within it, and the frequency with which these needs were identified across the 118 records.

The most frequently mentioned need was academic support and flexibility, investigated in 48 of 118 records, emphasizing adaptable learning structures that cater to individual needs. Andrews et al. (2020) found that targeted academic support, such as the Longhorn Opportunity Scholars (LOS) program, significantly increased college enrollment (by 71%) and long-term earnings (by 4.6%). Similarly, Noble et al. (2007) showed that faculty support, intellectual stimulation, and peer networks helped early university entrants succeed. Conejeros-Solar and Gómez-Arízaga (2015) examined academic support in gifted college students, finding that they faced greater challenges in faculty relationships, time management, and study habits, reinforcing the need for structured academic support.

Mentorship and career guidance, identified in 41 of 118 records, addresses a key need among students with high abilities for role models and direction to support their academic and professional success. Research highlights that career certainty and structured mentorship programs play a crucial role in meeting this need. Lunsford (2011) found that greater career certainty strengthened the quality of mentor relationships, suggesting that clear academic and professional goals enhance the benefits of mentorship. Mianehsaz et al. (2022) found that mentoring programs significantly increased academic motivation and research participation, confirming that structured guidance helps students stay engaged and excel. Similarly, Noble and Childers (2008) emphasized that mentorship is particularly essential for early university entrants, as these students rely on faculty mentorship, peer support, and structured academic transitions to navigate their accelerated academic paths successfully.

Counseling and mental health support, investigated in 35 records, relates to the need to address emotional well-being alongside academic achievement. Van den Muijsenberg et al. (2021) evaluated a group counseling program for underperforming students with high abilities, finding it effective in addressing key academic and personal challenges. Yusmansyah et al. (2019) found that religious counseling helped students with high abilities develop emotional strength and motivation. Watters (2010) emphasized the importance of career counseling, showing that teacher guidance and support influence students with high abilities’ professional aspirations.

#### 3.3.3. Teacher Training and Pedagogical Expertise

**Figure 7:**
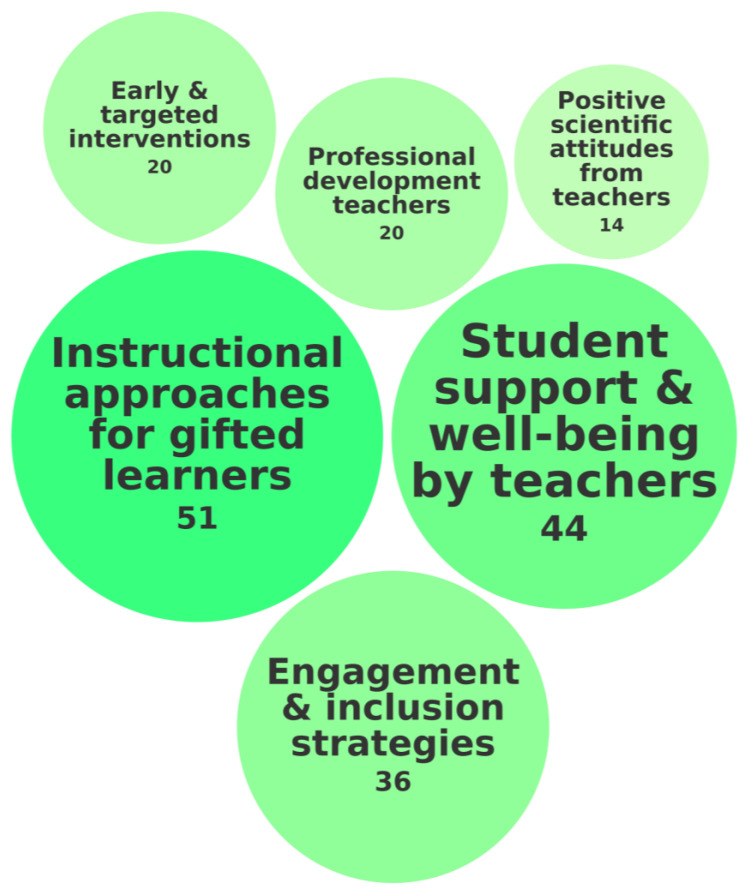
Teacher Training and Pedagogical Expertise: specific needs within this category and their frequency in 118 records.

The third category of needs for students with high abilities is Teacher Training and Pedagogical Expertise. It concerned equipping educators with the knowledge and skills necessary to provide effective support. Figure 7 illustrates this category of needs, the specific needs encompassed within it, and the frequency with which these needs were identified across the 118 records.

A key need within this category was instructional approaches tailored for gifted learners, investigated in 51 of 118 records. Research highlighted several challenges that reinforce this need. For example, Bain et al. (2006) found that misconceptions about giftedness among undergraduate students contribute to stigmatizing perceptions, emphasizing the necessity of educational programs to foster accurate understanding. Almukhambetova and Hernandez-Torrano (2021) examined the transition challenges faced by students with high abilities entering university, underscoring the importance of tailored instructional strategies to ease academic and social adaptation. Similarly, Gojkov et al. (2015) found that master’s students with high abilities lacked opportunities for critical thinking and independent learning, highlighting the need for problem-based learning and creative instructional methods to better support their intellectual development.

Student support and well-being by teachers, investigated in 44 records, underscores the critical role of educators in actively creating a supportive and emotionally nurturing learning environment. Teachers should not only focus on academic instruction but should also recognize and address students’ emotional and psychological needs. This includes fostering positive teacher–student relationships, promoting self-awareness and self-regulation, and encouraging peer connections. For example, Beduna and Perrone-McGovern (2016) found that emotional intelligence plays a key role in mediating the relationship between intellectual and emotional overexcitabilities and subjective well-being, highlighting the need for teachers to cultivate emotional awareness and resilience in students. Pandya (2023) demonstrated that integrating reflective or spiritual practices can enhance self-regulation, peer relationships, and overall well-being in students with high abilities. Similarly, Robins and Beer (2001) found that self-enhancement bias was linked to declining self-esteem and academic disengagement, reinforcing the importance of balancing academic development with well-being initiatives.

Engagement and inclusion strategies, investigated in 36 records, emphasize the need for an inclusive classroom culture to ensure that all students, including students with high abilities, remain challenged and engaged. For example, Narikbaeva (2016) highlighted the need for developing professional giftedness, showing that structured pedagogical conditions enhance students with high abilities’ development. Tindage et al. (2024) explored memorable messages in shaping the academic experiences of students of color, finding that positive memorable messages acted as microaffirmations while negative memorable messages were perceived as racial microaggressions, impacting student confidence. Salem et al. (2022) examined cooperation and altruistic behavior in students with high abilities, finding that intervention programs promoting cooperation and altruism can enhance social and academic engagement.

### 3.4. Types of Interventions

Regarding interventions aimed at higher education students with high abilities (RQ2), the analyses revealed that 42 of 118 records focused on interventions specifically designed to meet their needs. Of these 42 intervention-focused studies, 38 were aimed at students, 3 at student advisors and 1 at teachers.

#### 3.4.1. Interventions Targeted at Students

In the reviewed studies, interventions for students with high abilities in higher education addressed both social–emotional and educational needs through various approaches. The following sections outline how these interventions targeted key challenges in these domains. While some interventions focused directly on students, aiming to enhance their emotional well-being, motivation, and academic engagement (e.g., Ruban & Reis, 2006; Snyder et al., 2014), others were designed to support educators and counselors in creating environments that foster student success (e.g., Van den Muijsenberg et al., 2021). Interventions aimed at students often sought to create learning environments that stimulate intellectual engagement while also addressing emotional and psychological barriers to achievement. Honors programs, for instance, were designed to provide academically challenging experiences, allowing students with high abilities to engage with complex material and develop advanced skills (e.g., A. L. Miller & Dumford, 2018; Sedykh et al., 2021). Other approaches, such as gamified learning systems, introduced elements of competition and rewards to enhance motivation, particularly for students who struggled with engagement (e.g., Tan et al., 2023). Counseling programs played a key role in supporting students’ emotional well-being by addressing self-esteem, self-regulation, and the psychological demands of high achievement (e.g., Van den Muijsenberg et al., 2021; Yusmansyah et al., 2019). Beyond these broad strategies, specific findings underscore the impact of targeted interventions. One study highlighted the long-term benefits of structured pre-college programs that provide additional support for students from underrepresented backgrounds, improving retention rates and increasing access to higher education (Clasen, 2006). Another specific finding related to alternative assessment methods. Derado et al. (2016) demonstrated that the Point Reward System encouraged continuous engagement and reduced performance anxiety by offering flexible grading systems tailored to students’ needs. These findings emphasize the importance of adaptable and student-centered strategies in fostering both academic and personal development among students with high abilities.

#### 3.4.2. Interventions Targeted at Student Advisors and/or Teachers

Interventions for student advisors and/or teachers, mentioned in four records, were designed to enhance their ability to support students with high abilities effectively. Mentorship training for educators aimed to foster stronger teacher–student relationships and provided both academic and social–emotional support specifically tailored to students’ needs (e.g., Mianehsaz et al., 2022; Lunsford, 2011). Riise et al. (2022) focused on an intervention for teachers, introducing role model initiatives in STEMM fields to inspire students and meet their educational needs.

### 3.5. Needs Addressed by Interventions

#### 3.5.1. Needs Addressed by Interventions Targeting Students

Of the 38 identified records focusing on student interventions, quantifying the number of needs addressed in each intervention is essential for understanding the comprehensiveness and effectiveness of these approaches (RQ3). This analysis helps determine whether interventions are well-rounded or overly specialized, ensuring that students receive balanced support in both academic and social–emotional domains.

Among these 38 records, 10 addressed the full top three categories of social–emotional needs as well as the full top three categories of educational needs (Kerr & Kurpius, 2004; Lee et al., 2021; Liu, 2014; Mahenthiran & Rouse, 2000; Rinn & Boazman, 2014; Sedykh et al., 2021; Sloan, 2018; Tan et al., 2023; Thiemann, 2022; Van den Muijsenberg et al., 2021). However, none of the records addressed all three of the most frequently mentioned needs within these categories. Seven records covered five of the six categories of social–emotional and educational needs (Bain et al., 2010; Clark et al., 2018; Cross et al., 2018; Eiselen & Geyser, 2003; Martindale & Hammons, 2013; A. L. Miller et al., 2012; Ridgley et al., 2022).

Among interventions specifically targeting honors programs, two records addressed the full top three categories of social–emotional needs as well as all top three categories of educational needs (Lee et al., 2021; Sedykh et al., 2021). The most frequently addressed category of social–emotional needs in student interventions was the category Self-Perception, Motivation, and Performance Expectations. Needs in this category were investigated in 28 of the 38 records. The second most frequently addressed category was Social Integration and Acceptance of which the needs were reported in 23 of the 38 records. The third category, i.e., Emotional Regulation and Psychological Well-Being, was covered by 18 records. These findings highlight the predominant focus on academic motivation while indicating that emotional regulation and psychological well-being, though recognized, were less frequently emphasized in interventions.

#### 3.5.2. Needs Addressed by Interventions Targeting Student Advisors and/or Teachers

Of the three records focusing on interventions for student advisors, two addressed all top three categories of social–emotional needs as well as all top three categories of educational needs (Canaan et al., 2022; Yusmansyah et al., 2019). One record targeted an intervention for teachers (Riise et al., 2022), addressing five out of the six categories of social–emotional and educational needs. One category was not covered in this intervention, namely Teacher Training and Pedagogical Expertise.

### 3.6. Impact of Interventions on Reduction of Underachievement

In addressing the final research question (RQ4) on the impact of interventions, five out of the 118 records specifically focused on reducing or preventing underachievement among students with high abilities. These interventions either demonstrated or suggested a positive impact on mitigating underachievement. The studies examined a variety of approaches, including pre-college support programs, alternative assessment methods, peer mentoring, gamified learning, and counseling, highlighting the diverse strategies used to support students with high abilities.

First, the pre-college intervention examined by Clasen (2006) successfully increased high school graduation and college enrollment rates, particularly for high-ability minority and low-income students. The findings highlighted the long-term benefits of structured pre-college programs in supporting students who might otherwise be at risk of underachievement. Second, Derado et al. (2016) investigated the Point Reward System, which is an alternative assessment method designed to encourage continuous participation and engagement by shifting the focus from high-stakes exams to incremental progress and reward-based learning. Unlike traditional assessments that primarily evaluate final outcomes, this Point Reward System provided ongoing reinforcement, helping students to stay motivated and reducing the anxiety associated with single high-impact evaluations. Third, Tan et al. (2023) explored the effectiveness of gamified learning, demonstrating that integrating game-based elements into the learning process led to increased motivation and participation among underachievers. The results suggested that gamification can be a valuable tool for enhancing student engagement and improving academic outcomes for struggling students (Tan et al., 2023). Fourth, the Biggest Loser Competition, analyzed by O’Shea and Bigdan (2008), focused on peer mentoring as a strategy to support first-year engineering students. Results showed that structured peer mentoring, combined with incentive-based learning, significantly improved academic performance, emphasizing the role of collaborative support systems in mitigating underachievement. Fifth, Van den Muijsenberg et al. (2021) investigated a group counseling intervention aimed at university students with high abilities. They found that counseling effectively addressed social–emotional and academic barriers, providing students with valuable coping strategies to navigate their academic environments. Many participants reported improvements in their study habits, increased motivation, and a stronger sense of belonging. Furthermore, Van den Muijsenberg et al. (2021) highlighted the importance of individualized support, recommending that students be referred to personalized intervention programs when a need for more intensive treatment is identified. Overall, the five studies demonstrated varying degrees of effectiveness in addressing underachievement. However, measuring the impact of these interventions was sometimes challenging due to the lack of a baseline measurement, making it difficult to determine the exact extent of improvement.

## 4. Discussion

The current review study aimed to identify and map the social–emotional and educational needs of higher education students with high abilities, analyze the types of interventions implemented, and evaluate the extent to which these interventions address the identified needs and reduce underachievement. The findings highlight the complexity and diversity of needs among students with high abilities, the varied approaches taken to support them, and the gaps in current intervention strategies. While several effective interventions exist, their comprehensiveness remains uneven, with a stronger focus on motivation and academic development but less attention given to emotional regulation, stress management, and educator training.

First, the current review study identified 20 social–emotional needs and 26 educational needs, which were categorized into six overarching domains. Self-perception, Motivation and Performance Expectations emerged as the most frequently addressed category of social–emotional needs, underscoring the importance of self-confidence, perseverance, and ambition in sustaining academic engagement (Al-Shabatat et al., 2010; Tsai & Fu, 2016). Additionally, a need for support and understanding were identified as key factors in student well-being, influencing academic persistence and overall success (Baslanti & McCoach, 2006). However, emotional regulation and psychological well-being received significantly less attention, despite their known role in preventing burnout and fostering long-term engagement (Cross et al., 2018; Ridgley et al., 2022). In the domain of educational needs, the highest priority was given to the category Curriculum Design and Instructional Strategies, emphasizing the value of personalized learning, differentiation, and self-directed study approaches (Noble & Childers, 2008; Griffioen et al., 2018). Structured learning environments were recognized for providing clear guidance and academic support, while self-directed learning methods were seen as essential for fostering autonomy, problem-solving skills, and a sense of ownership over one’s learning process (Woody & Parker, 2012). Despite these advancements, a more holistic approach to educational needs is necessary, ensuring that students with high abilities receive both the academic challenge and emotional support required for their development.

Second, an analysis of interventions revealed a predominant focus on student-centered approaches, with far fewer initiatives targeting student advisors and teachers. While programs such as honors courses, gamified learning, and counseling services have been widely implemented to enhance academic engagement and social–emotional well-being (A. L. Miller & Dumford, 2018; Sedykh et al., 2021; Tan et al., 2023; Van den Muijsenberg et al., 2021), the lack of faculty development efforts presents a critical gap in existing support structures. Interventions directed at student advisors were particularly scarce, with only two studies (Canaan et al., 2022; Yusmansyah et al., 2019) addressing both social–emotional and educational needs. Given that advisors play a key role in guiding students with high abilities through academic and personal challenges, the absence of comprehensive training programs limits their effectiveness. More effort is needed to integrate advisor-focused interventions with student-centered strategies to create a more holistic support system (Mianehsaz et al., 2022).

Even more concerning is the near absence of teacher-focused interventions, with only one study (Riise et al., 2022) explicitly addressing their role, despite instructional strategies for students with high abilities being one of the most frequently identified educational needs. Teachers are responsible for not only delivering high-quality instruction but also fostering students’ social–emotional well-being and motivation, both of which are critical for academic success. The lack of research on how best to equip teachers with the necessary skills to support students with high abilities represents a major oversight. While much attention has been given to student-directed interventions, neglecting teacher preparation limits the potential for sustainable, system-wide improvements. Expanding teacher-focused interventions is essential to ensure that faculty are adequately prepared to meet the diverse needs of students with high abilities, ultimately bridging the gap between research and effective classroom practice.

In this context, the importance of pre-service teacher training should not be overlooked. Although this review focused on students currently enrolled in higher education, several studies (e.g., Erdogan, 2017; Avsec & Savec, 2021) point to the critical role of pre-service training in equipping future teachers with the pedagogical competencies needed to recognize and support giftedness. These findings suggest that investing in teacher preparation at all stages can contribute to long-term improvements in instructional quality and help address systemic barriers to meeting the needs of students with high abilities in higher education.

Third, findings concerning the extent to which interventions addressed student needs reveal strengths and gaps in current strategies. While some interventions covered all categories of social–emotional and educational needs (Kerr & Kurpius, 2004; Lee et al., 2021; Sedykh et al., 2021; Van den Muijsenberg et al., 2021), no intervention addressed all frequently mentioned needs comprehensively. The strongest emphasis was on the category Self-Perception, Motivation and Performance Expectations, with 28 records focusing on this area (Rinn & Boazman, 2014; Tan et al., 2023). This aligns with previous research indicating the centrality of motivation in academic success. However, emotional regulation and psychological well-being were less frequently addressed, despite their known impact on student retention and academic performance (Clark et al., 2018). Counseling and mentoring interventions targeted self-esteem and emotional regulation, fostering resilience, though their direct impact on academic performance remained unclear (e.g., Van den Muijsenberg et al., 2021). While some programs provided promising frameworks for coaching students with high abilities (e.g., Lunsford, 2011; Van den Muijsenberg et al., 2021), it can be concluded that there is a need for more comprehensive training programs that equip both teachers and student advisors with the necessary skills to support students with high abilities complex and diverse needs. Addressing these gaps is essential to ensuring the success of those students in diverse and evolving educational contexts.

Fourth, an analysis of the interventions aimed at reducing underachievement underscores the potential of targeted support strategies in fostering academic success and engagement. While diverse approaches have been explored, pre-college programs, alternative assessments, gamification, peer mentoring, and counseling have demonstrated varied levels of effectiveness. Pre-college programs were particularly effective in supporting minority and low-income students, increasing graduation and college enrollment rates (Clasen, 2006), while alternative assessments, such as the Point Reward System, helped reduce anxiety and sustain engagement (Derado et al., 2016). Similarly, gamification and peer mentoring have shown promise in enhancing motivation and persistence, reinforcing the importance of collaborative and interactive learning environments (Tan et al., 2023; O’Shea & Bigdan, 2008). Counseling further addressed both academic and emotional barriers, supporting students in developing resilience and effective study habits (Van den Muijsenberg et al., 2021). Despite these promising outcomes, the impact of interventions remains difficult to measure, as some studies lacked baseline data and long-term follow-up, limiting the ability to assess sustained effectiveness. Future research should focus on longitudinal studies and control-group comparisons to provide more robust evidence of what works best for students with high abilities at risk of underachievement. Ultimately, a comprehensive, multi-level approach, integrating academic, social–emotional, and psychological support, is essential to ensuring long-term engagement and success for this student population.

### 4.1. Strengths, Limitations and Future Research

The current review study has several strengths. For example, it concerns an extensive, structured and comprehensive systematic review approach. Furthermore, it followed an OSF pre-registered screening process that aligned with PRISMA statement guidelines (Moher et al., 2010). Additionally, the review synthesized findings across a diverse range of studies, offering a broad yet detailed perspective on both social–emotional and educational needs, as well as interventions aimed at addressing underachievement among students with high abilities in higher education.

However, several limitations should be considered. First, a key challenge in this review is the variability in defining high ability, as studies use different criteria (e.g., IQ thresholds, academic performance, domain-specific talents), introducing bias in comparisons and synthesis. Second, there is a publication bias since the current review included only peer-reviewed, English-language studies from four databases, potentially excluding relevant research published in other languages and/or accessible through other databases. Third, the large heterogeneity in used research methods, sample sizes, and types of interventions limited the comparisons and generalizability, and the absence of standardized outcome measures complicated synthesis. Fourth, the time lag in evidence, referring to the gap between when studies were conducted and when their findings are applied, may impact their current relevance. Educational practices, student needs, and institutional policies evolve over time, meaning that older studies may not fully reflect the present-day challenges and opportunities in higher education. Additionally, both selection bias and the lack of baseline measurements in some interventions made impact assessment challenging in the current review study, as it was difficult to determine the precise effectiveness of interventions without a clear pre-intervention comparison. Finally, integrating qualitative and quantitative data presents interpretation difficulties. Despite these limitations, the review highlights key trends, identifies research gaps, and outlines future directions.

Building on the findings of the current review study, several key areas for future research emerge. While the need for effective interventions to reduce underachievement among students with high abilities is relevant across the entire education field, the current study specifically focused on higher education. Despite growing awareness of this issue, many questions remain unanswered. One critical area is teacher-focused interventions, as professional development programs that equip educators with differentiated instructional strategies and methods to foster student motivation are essential for supporting students with high abilities. Effective teaching approaches can enhance engagement, prevent underachievement, and ensure that students receive the appropriate level of challenge and support. However, as this review has shown, research in this area remains limited, revealing a significant gap that must be addressed. This gap presents an opportunity for curriculum developers and teacher professionalization programs to strengthen instructional practices in higher education. Future research should examine effective professional development models, assess the long-term impact of teacher-focused interventions on student outcomes, and explore the practical application of instructional differentiation across disciplines. Additionally, examining how teachers perceive and implement these strategies in practice would provide valuable insights into optimizing and sustaining teacher-focused interventions for students with high abilities.

Another key question for future research is how the needs of students with high abilities in higher education vary across different fields of study. Academic disciplines present unique challenges and opportunities, shaping students’ learning experiences, motivation, and support requirements. For instance, STEM disciplines often emphasize independent research, hands-on experimentation, and specialized mentorship (e.g., Riise et al., 2022; Merzon et al., 2013), while humanities and social sciences tend to focus on discussion-based learning, critical thinking, and interdisciplinary exploration (e.g., Sedykh et al., 2021). Additionally, disciplines with highly structured curricula follow a rigid, sequential progression with clearly defined coursework, offering little flexibility for individualized learning paths. In contrast, more open-ended disciplines provide students with greater autonomy in course selection but may lack clear academic direction, requiring a more self-directed approach to structuring their education. Investigating how these differences impact engagement, achievement, and well-being among students with high abilities could provide valuable insights for designing more effective, discipline-specific support systems.

Continuing with recommendations for future research, an important gap in the reviewed studies is the lack of a systematic examination of whether the needs of students with high abilities vary by field of study. Although the studies included students from various academic programs, none specifically analyzed discipline-specific challenges and their impact on underachievement. This limits the understanding of how different academic contexts shape students’ experiences and support needs. Future research should aim to bridge this gap by conducting comparative studies across disciplines, identifying whether tailored interventions are necessary for different academic fields.

Lastly, future studies should also explore how institutional policies and structural support systems can create inclusive learning environments that cater to diverse students, including those with high abilities. Institutional commitment to recognizing and addressing the needs of students with high abilities is essential for fostering an equitable and supportive academic environment. By addressing the perspectives of both students and educators, future interventions should prioritize a collaborative approach, ensuring sustainable academic and social–emotional success. Additionally, examining the way policies regarding flexible learning pathways, faculty training, and student support services impact students with high abilities could provide actionable recommendations for higher education institutions seeking to optimize their support systems.

### 4.2. Conclusions

The findings of the current review study emphasize the necessity of integrated support systems for students with high abilities in higher education and the importance of recognizing the interplay between academic challenge, emotional well-being, social interaction, and instructional effectiveness in optimizing these students’ outcomes. It is essential to develop a comprehensive and interdisciplinary research agenda to stimulate evidence-based practices that support these higher education students with high abilities. Furthermore, a holistic approach is key with interventions that integrate educational needs (such as the opportunity to develop and effective instruction) with social–emotional support, ensuring that students with high abilities receive comprehensive guidance to foster both academic excellence and personal well-being. By addressing these interconnected needs simultaneously, interventions can create learning environments that promote engagement, motivation, and resilience, ultimately reducing underachievement and maximizing student potential.

## Figures and Tables

**Table 1:** Records included in the systematic review with authors, country, research institution, study design, sample size, instruments, and findings.

Authors	Country	Research Institution	Study Design	Sample Size	Instruments	Findings
Afshar and Movassagh (2017)	Iran	A public university	Survey	76	California Critical Thinking Skills Test (CCTST), Strategy Inventory for Language Learning (SILL)	Critical thinking and strategy use had a significant positive correlation with university achievement, with critical thinking being stronger and an asset to the high-achieving group.
Afshari et al. (2020)	Iran	A public university	Mixed method	*n*_control_ = 30 *n*_experimental_ = 30	Strategy Inventory for Language Learning (SILL), International English Language Testing System (IELTS)	Group dynamic assessment was more effective than conventional explicit intervention for supporting EFL writing development, and it worked best for low-ability learners. In addition, this intervention promoted both EFL writing and learner self-regulation.
Al-Khayat et al. (2017)	Jordan	A public college	Survey	66	Questionnaire developed by the authors	The highest teaching strategies preferred by gifted students were related to the creative thinking dimension followed by critical thinking strategies and accommodations for individual differences, with the lowest being presentations.
Al-Qahtani (2021)	Saudi Arabia	A non-specified university	Survey	92	Test of English as Foreign Language (TOEFL), Survey of Reading Strategies (SORS)	Each ability level reported strategy was used differently in terms of order and intensity. There was also a statistical significance in strategy use between the high ability and the low ability levels. The low ability-level participants reported higher use of the global reading strategies than the high-ability group.
Al-Shabatat et al. (2010)	Malaysia	A non-specified university	Survey	180	Cattell Culture Fair Test (CCFT)	Achievement motivation and fluid intelligence significantly influenced intellectual giftedness, with strong direct and indirect effects. Motivation factors like self-confidence, perseverance, and autonomy enhanced cognitive growth, supporting the development of giftedness.
Al-Ta’ani and Hamadneh (2023)	Jordan	A private university	Survey	86	Questionnaire developed by the authors	Active learning strategies enhanced students’ motivation and encouraged faculty to foster and stimulate students’ enthusiasm for learning.
Ali et al. (2021)	Pakistan	A public university	Survey	180	Self-Report Measure of Emotional Intelligence (SRMEI)	Male students exhibited higher emotional intelligence than female students, particularly in the areas of emotional self-regulation and emotional self-awareness. However, no significant gender difference was found in interpersonal skills.
Almousa et al. (2022)	Jordan	A public and a private university	Survey	353	Questionnaire developed by the authors	Academic challenges were the biggest concern for high-achieving students, while family issues had minimal impact. Bachelor’s students struggled more than advanced-level students, suggesting a need for early academic support. Females experienced more emotional distress, highlighting the need for mental health interventions. STEM students faced more study-related issues, requiring targeted academic support.
Almukhambetova and Hernandez-Torrano (2021)	Kazakhstan	Two universities	Mixed method	201	Student Adaptation to College Questionnaire (SACQ)	The adjustment of gifted students to university was found to be a complex process, requiring a comprehensive consideration of freshmen experiences when examining their transition to post-secondary education.
Andrews et al. (2020)	US	Diverse institutions	Survey	>1,000,000	Data analysis	Longhorn opportunity scholars’ program had large, positive effects on enrolment in and graduation from UT-Austin, master’s degree enrolment, and earnings.
Ashtiyani et al. (2013)	Iran	A public university	Survey	*n*_control_ = 180 *n*_experimental_ = 56	Questionnaire developed by the authors	The mean score of educational, research, and psycho-spiritual problems between gifted and ordinary students differed significantly (*p* > 0.05). The multivariate regression model predicted 52.8 of the total variances of the gifted students’ problems.
Avsec and Savec (2021)	Slovenia	A public university	Survey	225	Questionnaire developed by the authors	Enhancing the transformative aspect of education for sustainable development in pre-service teachers required critical reflection, self-awareness, risk-taking, a holistic perspective, openness to diversity, and social support. Additionally, self-directed learning played a moderating role in transformative learning among pre-service science teachers.
Bain et al. (2006)	US	Not specified	Survey	285	Questionnaire developed by the authors	Identified misconceptions about homogeneity, synchronous development, and emotional/social distress in gifted children and their non-gifted siblings.
Bain et al. (2010)	US	A public university	Survey	88	Foreign Language Attitudes and Perceptions Survey-College (FLAPS-C), Modern Language Aptitude Test (MLAT), College Academic Attribution Scale- Foreign Language (CAAS-FL)	Gifted students scored higher in aptitude and had a more positive attitude toward learning a foreign language, but both groups showed no differences in success attributions.
Barth et al. (2018)	US	A non-specified college	Survey	526	Questionnaire developed by the authors	Students held stereotypical ability beliefs of others but showed no gender differences in STEM self-efficacy or career interests.
Baslanti and McCoach (2006)	Turkey	A public university	Survey	165	School Attitude Assessment Survey-Revised (SAAS-R)	Five key factors of underachievement were identified: academic self-perceptions, attitudes toward teachers, attitudes toward school, goal valuation, and motivation/self-regulation with motivation/self-regulation as the strongest predictor.
Bauer et al. (2023)	Germany	Diverse institutions	Mixed method	3584	Questionnaire developed by the authors	First-generation students perceived themselves as less talented but equally diligent, which impacted their academic experience and engagement. This self-concept bias was strongest in talent-focused environments but lessened when effort was emphasized.
Beduna and Perrone-McGovern (2016)	US	A public university	Survey	144	Overexcitabilities Questionnaire II (OEQII), Satisfaction With Life Scale (SWLS), Brief Emotional Intelligence Scale (BEIS-10)	Emotional and intellectual overexcitability were positively linked to emotional intelligence, which in turn was positively associated with subjective well-being. Path analysis confirmed that emotional intelligence mediated the relationship between overexcitability and well-being.
Bell and McCallum (2012)	US	A non-specified university	Survey	95	Modern Language Aptitude Test (MLAT), Questionnaire developed by the authors, Foreign Language Attribution Scale (FLAS), Foreign Language Attitudes and Perceptions Survey (FLAPS)	Modern language aptitude test part IV and luck attributions significantly predicted exam grades within a multiple regression analysis. In a second multiple regression analysis, only effort and ability attributions significantly predicted anxiety.
Ben-Eliyahu (2019)	US	A non-specified university	Mixed method	271	Questionnaire developed by the authors, Patterns of Adaptive Learning Scales (PALS), Academic Focusing Scale (AFS), Self-Control scale, Social Achievement Goals Scale, Motivated Strategies for Learning Questionnaire (MSLQ)	Gifted students exhibited diverse self-regulated learning (SRL) profiles, with three groups: highly regulated, regulated, and behaviorally dysregulated. Typical students most resembled the regulated group. Behaviorally dysregulated gifted students had lower academic and social SRL but showed no differences in trait emotion regulation or self-control, suggesting a situated SRL effect. Gifted students also showed stronger links between motivation and SRL, with mastery goal orientation predicting all SRL forms only for them.
Bennett (2009)	UK	A non-specified university	Survey	511	Questionnaire developed by the authors	Three major facets of academic self-concept were found to be particularly relevant: self-belief in one’s academic competence, self-appreciation of one’s personal worth as a student, and self-connection with being an undergraduate.
Bergold et al. (2021)	Germany	Not specified	Survey	*n*_control_ = 87 *n*_experimental_ = 345	Experiment designed by the authors	Stereotypical representations in the media about intellectually gifted individuals contributed to the stigmatization of gifted individuals. Nonstereotyped, evidence-based representations caused more positive attitudes.
Boazman and Sayler (2011)	UK	A public university	Survey	157	General Perceived Self-Efficacy Scale (GSE), Questionnaire developed by the authors, Personal Well-being Index-Adult (PWI-A), State Trait Cheerfulness Inventory (STCI-T)	Early college entrants expressed greater global satisfaction with their lives than older peers. They reported elevated levels of satisfaction in their achievements, immediate standard of living, personal safety, and future security than age peers. They expressed powerful feelings of general self-efficacy and high levels of trait seriousness, which are two constructs related to facilitating success.
A. Campbell (2019)	South Africa	A public university	Mixed method	265	Mindset Assessment Profile Tool	Without experiencing interventions aimed at developing growth mindset, students showed small shifts towards stronger growth mindsets over their first year.
K. C. Campbell and Fuqua (2008)	US	A public university	Survey	336	Attitude toward Ability Grouping Questionnaire	The most important discriminating variables predicting student completion were high school GPA, high school class rank, first semester college GPA, gender, and initial housing assignment (honors housing or other).
Canaan et al. (2022)	Lebanon	A private university	Survey	3857	Data analysis	Higher adviser value-added significantly improved freshmen GPA, time to completion, and four-year graduation rates. Additionally, it increased the likelihood of high-ability students enrolling in and graduating with a STEM degree.
Caplan et al. (2002)	US	A public college	Survey	162	Student Adaptation to College Questionnaire (SACQ), Self-Report Measure of Emotional Intelligence (SRMEI), Overexcitabilities Questionnaire II (OEQII)	Measures of student self-concept and family environment provided valuable performance and adjustment insights for early entrance programs. Identifying at-risk students and families could help with academic and retention challenges, while programs supporting emotional growth and personal adjustment could benefit all admitted students.
Clark et al. (2018)	US	A non-specified university	Survey	393	Transition to College Inventory (TCI)	Honors college students achieved high grades and retention rates, with self-confidence and external influences on college choice as key non-cognitive predictors of these outcomes.
Clasen (2006)	US	A public university	Mixed method	158	Questionnaire developed by the authors and interviews	The findings supported the value of multiple identification methods, including problem-solving, teacher-identified leadership, and GPA. Additionally, a long-term university/school partnership program for underrepresented gifted students showed that higher student involvement correlated with better academic outcomes.
Conejeros-Solar and Gómez-Arízaga (2015)	Chile	A semi-public university	Mixed method	254	Student Adaptation to College Questionnaire (SACQ), Questionnaire developed by the authors	Gifted students showed stronger academic development and faculty relationships in college. However, they struggled with time management, weak study habits, and gaps in content knowledge due to poor high school academic preparation.
Cross et al. (2018)	US	A non-specified university	Survey	410	Questionnaire, Big Five Inventory (BFI), Adult Suicidal Ideation Questionnaire (ASIQ)	High perfectionism and suicidal ideation were risk factors in two student profiles (possible misfits and serious students), suggesting a need for enhanced psychological support. The largest group (typical friendly) had above-norm extraversion, while all other profiles had below-norm introversion. Neuroticism was higher than the norm in introverted profiles.
Deibl and Zumbach (2023)	Austria	A non-specified university	Survey	156	Myths and Facts Questionnaire, SESSKO Questionnaire	Results showed no significant difference between freshmen and advanced students in identifying myths, but freshmen identified slightly more facts correctly. Self-confidence was crucial, as master’s students with high self-confidence identified more facts correctly.
Derado et al. (2016)	US	A public university	Survey	976	Point Reward System (PRS), Traditional assessment method, Student Impact Index (SII)	The study found that the Point Reward System significantly lowered withdrawal, failure, and dropout rates, improved student engagement, and had a greater impact on learning compared to traditional assessment methods in college mathematics classrooms.
Domínguez-Soto et al. (2023)	Spain	A non-specified university	Survey	584	Multifactor Leadership Questionnaire (MLQ), Clance Impostor Phenomenon Scale (CIPS), Harvey Impostor Scale, Perceived Fraudulence Scale, Leary Impostor Scale, IPP-31	The CIPS demonstrated strong reliability (α = 0.826), supporting its sensitivity and reliability in measuring impostor syndrome. The MLQ was reported as well-established, with previous research indicating acceptable reliability in European samples (ranging from 0.61 to 0.78). However, in this study, some factors had lower reliability (as low as 0.47), which led the researchers to simplify the model to three leadership factors.
Eddy et al. (2013)	US	A public university	Survey	817	Experiment designed by the authors	Students who built their own phylogenetic trees performed significantly better in assessments than those who analyzed existing trees. Undergraduate students who completed a tree building activity scored similarly to Biology PhD students, suggesting its effectiveness in teaching tree-thinking.
Eiselen and Geyser (2003)	South Africa	A public university	Mixed method	45	Questionnaire developed by the authors, Brown Holzman Survey of Study Habits and Attitudes (SSHA C), General Scholastic Aptitude Test (GSAT)	Achievers demonstrated better communication skills, diligence, and cognitive abilities than at-risk students, earning higher school marks. Additionally, their perceptions of success and failure differed significantly from those of at-risk students.
Ellala et al. (2022)	United Arab Emirates	Not specified universities	Survey	388	Questionnaire developed by the authors	Among UAE students, enthusiasm and agility positively correlated with linguistic ability. For gifted students, institutional support significantly enhanced attention, skill, and linguistic intelligence.
Elsamanoudy and Abdelaziz (2020)	United Arab Emirates	A private university	Survey	130	Questionnaire developed by the authors	The study found that 66% of interior design students joined due to passion, and 95% effectively learned project requirements. Teaching strategies had a significant positive impact on third-year students. The study concluded that creative and critical thinking strategies should replace conventional methods like memorization to help gifted students reach equal motivation levels as their peers.
Erdogan (2017)	Turkey	A public university	Survey	82	Science Teaching Attitude Scale, Scientific Attitude Inventory (SAI II)	A significant grade-level difference and a strong correlation between scientific attitudes and science teaching attitudes was found. It recommends creating learning environments that positively influence both attitudes.
Fong and Krause (2014)	US	A public university	Mixed method	49	Nelson–Denny Reading Test (NDRT)	Underachievers had significantly less mastery experiences and verbal persuasions despite having similar levels of self-efficacy.
Gojkov et al. (2015)	Serbia	A public research university	Survey	112	Questionnaire developed by the authors Didactic strategies and competencies of gifted students (DSCGS-1)	The highest-achieved competencies were crucial for intellectual functioning but not directly linked to critical thinking, intellectual autonomy, or deep conceptual understanding. They were more related to basic knowledge, factual understanding, and event explanations.
Griffioen et al. (2018)	The Netherlands	A public college	Survey	733	Questionnaire developed by the authors	Cognitively abler students were less satisfied in vocational education, feeling insufficiently challenged cognitively and creatively and harder to satisfy.
Heilbronner (2011)	US	Not specified	Survey	360	Questionnaire developed by the authors The Pathways Survey	Key predictors of choosing a STEM major in college were self-belief in STEM ability and the quality of academic experiences, including challenge level, hands-on learning, and career preparation adequacy.
Heilbronner et al. (2010)	US	A private university	Mixed method	43	Questionnaire developed by the authors PEG Alumnae Survey	Students who left an early college acceleration program often sought greater academic challenges or specialized majors, supporting the idea of positive attrition.
Hevel et al. (2015)	US	Diverse institutions	Survey	2212	Secondary data analysis, Critical Thinking Test (CTT), National Survey of Student Engagement (NSSE), WNS Student Experiences Survey, Defining Issues Test 2 (DIT2), Need for Cognition Scale (NCS), Positive Attitude Toward Literacy Scale (PATLS), Ryff Scales of Psychological Well-Being (RPWB)	The study found no direct effect of fraternity/sorority membership on educational outcomes in the fourth year of college. However, it identified five conditional effects related to students’ academic abilities and racial/ethnic identities. Fraternity/sorority membership negatively affected critical thinking in White students but had no effect on students of color. It was also associated with lower moral reasoning in students of color but higher in White students. Additionally, students with lower pre-college critical thinking skills experienced negative effects on critical thinking, while those with higher pre-college need for cognition showed growth.
Hoogeveen et al. (2012)	The Netherlands	A public research university	Survey	203	Questionnaire developed by the authors (incl. Self-Description Questionnaire SDQ) and observation	The study found minimal social–emotional differences between accelerated and non-accelerated gifted students, with small advantages for accelerated students. Multiple grade skipping had no negative effects, and long-term outcomes of acceleration were positive. Personal and environmental factors only impacted non-accelerated students.
Ibragimova and Ponomareva (2020)	Russia	A public research university	Survey	166	Questionnaire developed by the authors	Modern methodological tools and digital platforms played a crucial role in enhancing legal education for gifted students. Future law teachers’ pedagogical competencies were essential for effectively utilizing these tools, and digitalization improved educational environments for gifted students.
Kerr and Kurpius (2004)	US	Not specified	Survey	502	Vocational Preference Inventory (VPI), Adolescent At-Risk Behaviors Inventory (AARBI), Career Behaviors Inventory (CBI), Educational Self-Efficacy-Adolescence Scale (ESEA), Rosenberg Self-Esteem Scale (RSES)	Self-esteem, school self-efficacy, and future self-efficacy increased from pre-test to follow-up. Girls showed greater career exploration and were more likely to persist in nontraditional career choices.
Kokkinos and Gakis (2021)	Greece	A public university	Mixed method	142	Questionnaire developed by the authors and observations	Participants primarily differentiated teaching based on students’ learning readiness but provided less support for high-achievers, mainly by offering more difficult tasks and higher-order thinking activities. They believed their strategies mainly influenced students’ cognitive learning.
Lakin and Wai (2020)	US	Diverse institutions	Survey	506,984	Secondary data analysis	Spatially talented students faced greater academic challenges, including reading difficulties, poor study habits, and behavioral issues, and were less likely to complete college degrees than other talented students.
Lapointe-Antunes and Sainty (2023)	US	A public research university	Mixed method	360	Secondary data analysis Questionnaire developed by the authors	An immersive case improved student practice performance but not specifically for high-ability students. Extensive six-to-eight-week exam preparation helped close performance gaps among students.
Lee et al. (2021)	US	A non-specified university	Survey	244	Clance Impostor Phenomenon Scale (CIPS), Multidimensional Perfectionism Scale (MPS)	Socially prescribed perfectionism and honors program participation were associated with higher imposter feelings in undergraduate students.
Li et al. (2023)	China	Two universities	Survey	818	Questionnaire, Nonrestorative Sleep Scale (NRSS), Perceived Stress Scale (PSS-10), Connor–Davidson Resilience Scale (CD-RISC-10), Kessler Psychological Distress Scale (K10)	Freshmen students exhibited heterogeneity in perceived stress, which mediated the link between nonrestorative sleep and emotional distress. However, resilience did not significantly moderate these relationships.
Limeri et al. (2023)	US	Diverse institutions	Survey	1194	Undergraduate Lay Theories of Abilities Survey (ULTrA)	Mindset, brilliance, and universality were distinct and empirically discriminable constructs rather than aspects of the same belief. Using the Undergraduate Lay Theories of Abilities survey, factor analyses, and Structural Equation Models showed that each belief uniquely influenced psychosocial and academic outcomes.
Liu (2014)	Taiwan	A non-specified university	Survey	143	Foreign Language Classroom Anxiety Scale (FLCAS), Attitude toward Ability Grouping Questionnaire	High-achieving students experienced higher anxiety in English classes, particularly through tension in class, nervousness when speaking, and fear of being laughed at. Students generally favored ability grouping, believing it benefited their language learning.
Lubinski et al. (2001)	US	Diverse institutions	Survey	1470	Scholastic Assessment Test (SAT), Study of Values (SOV), Strong Vocational Interest Inventory (SVII)	World-class U.S. math–science graduate students exhibited exceptional quantitative reasoning, strong scientific interests, and persistence in scientific skill development from adolescence. They were identifiable early based on non-intellectual attributes, aligning with profiles of distinguished scientists. Sex differences were minimal among graduate students but present in the comparison group. Developing scientific expertise required similar educational experiences for both sexes.
Lunsford (2011)	US	A not specified research university	Mixed method	128	Mixed method (interviews and multinomial logistic regression)	One-fourth of academically talented students did not feel mentored, and career certainty was linked to mentor relationship quality. As students’ career certainty increased, they reported better mentoring experiences.
Ma’ajini (2013)	Saudi Arabia	A public research university	Survey	260	Questionnaire developed by the authors	Career certainty significantly predicted mentor relationship quality. Students with developing career plans reported better mentoring experiences than those without career plans.
Mahenthiran and Rouse (2000)	US	A non-specified university	Survey	110	Job Diagnostic Survey	Students had more positive attitudes when allowed to choose a friend in their group. Group project grades were higher in paired assignments than in randomly formed groups.
Maranges et al. (2023)	US	Diverse institutions	Survey	467	Questionnaire developed by the authors	Brilliance beliefs predicted women’s but not men’s choice of major. Women with lower brilliance beliefs were more likely to choose psychology over philosophy. Mindset (fixed vs. growth) did not differ by gender or influence major choice. Findings suggest that internalized gendered beliefs about brilliance shape academic field selection.
Martindale and Hammons (2013)	US	A public research university	Mixed method	1761	Questionnaire developed by the authors Data analysis	Mandatory interventions for students with low first-semester GPA’s led to higher final GPAs and increased scholarship renewal rates, while voluntary interventions were ineffective. Students felt these interventions showed university support and helped them retain scholarships.
Merzon et al. (2013)	Russia	A public research university	Survey	Not specified	Questionnaire Bennett Mechanical Comprehension Test, Amthauer Intelligence Structure Test (IST), Klimov’s DDC	Students in technical institutes demonstrated higher technical comprehension, spatial thinking, and imagination than children. The educational environment in technical institutes enhanced technical skills, leading to above-average achievements in construction and technology.
Mianehsaz et al. (2022)	Iran	A public university	Mixed method	21	Science Motivation Questionnaire II (SMQ-II)	Talented students performed well as mentors, and implementing a mentoring program boosted academic motivation and research engagement among undergraduate nursing students while also preventing GPA decline.
A. L. Miller and Dumford (2018)	US	Diverse institutions	Survey	8530	National Survey of Student Engagement (NSSE)	Honors college participation positively influenced reflective and integrative learning, learning strategies, collaborative learning, diverse discussions, student–faculty interaction, and quality of interactions for first-year students. For seniors, it was associated with more frequent student–faculty interaction. These findings highlight the experiential and curricular benefits of honors programs.
A. L. Miller et al. (2012)	US	A non-specified university	Survey	323	National Survey of Student Engagement (NSSE), Parenting Style Questionnaire, Scale of Creative Attributes and Behaviors (SCAB)	The study found positive relationships between permissive parenting and creativity and between authoritarian parenting and socially prescribed perfectionism. Negative relationships were observed between authoritarian parenting and creativity. Significant links were also identified between creativity and gender, as well as between parenting styles and creativity or perfectionism.
A. L. Miller et al. (2021)	US	Diverse institutions	Survey	1487	Faculty Survey of Student Engagement (FSSE)	Regression analyses revealed that faculty teaching honors courses were more likely to promote student–faculty interaction, learning strategies, and collaborative learning, even after accounting for demographic and institutional factors.
A. L. Miller and Smith (2017)	US	A non-specified university	Survey	387	Scale of Creative Attributes and Behaviors (SCAB)	The findings indicated significant differences by academic major in creative engagement, cognitive style, and fantasy, but not in tolerance or spontaneity. Arts and humanities majors generally demonstrated higher creativity compared to fields like education and pre-professional training, although all majors benefited from creative input.
C. J. Miller and Bernacki (2019)	US	A non-specified university	Mixed method	32	Self-Regulated Learning Training (SoL2L), Assessment and Learning in Knowledge Spaces (ALEKS)	SRL-trained students demonstrated greater learning efficiency and mastered math topics more effectively during digital problem-solving. A follow-up mixed methods case study based on the Situated Model of Self-Regulated Learning (SRL) highlighted adaptive learning strategies used by high-ability self-regulators, contrasting them with less effective approaches of untrained learners.
Narikbaeva (2016)	Kazakhstan	A public research university	Survey	851	Questionnaire developed by the authors Torrance’s Tests of Creative Thinking (TTCT), IQ test	Students’ IQ and creativity test results highlighted the need to enhance efforts in developing professional giftedness at the university. A comprehensive pedagogical approach was designed, addressing organizational, psycho-pedagogical, and didactic levels to support giftedness development.
Nazarova et al. (2018)	Russia	A public research university	Survey	62	Experiment designed by the authors	Scientific research work enhanced professional competence, creativity, and teamwork skills, engaged all students regardless of aptitude, and helped identify gifted, motivated individuals.
Noble and Childers (2008)	US	A public university	Mixed method	70	Questionnaire developed by the authors	All early entrants required intellectual preparation, peer groups, a supportive home base, mentoring faculty, and a welcoming environment. Younger students needed intensive academic transitioning, while older students required a balance of independence and guidance. Parental and institutional preparation were crucial for success at any age.
Noble et al. (2007)	US	A public university	Survey	95	Questionnaire developed by the authors	Respondents largely chose early university entrance for the excitement of learning and valued the peer group, intellectual stimulation, and faculty support. Some felt too young for major decisions, with males more often regretting the lack of dating opportunities due to age differences.
O’Conner (2003)	UK	Two universities	Mixed method	129	Questionnaire developed by the authors Interviews	Institutional conditions, including program design, resource quality, and the university environment, significantly impacted learning and motivation in universities with low-ability students. The three-phase mixed methods approach provided comprehensive insights, highlighting the need for targeted support and resource allocation to enhance student outcomes.
O’Shea and Bigdan (2008)	Australia	A public research university	Mixed method	*n*_control_ = 199 *n*_experimental_ = 146	Experiment designed by the authors with questionnaires, discussion forums and reflections	High-achieving students recognized that helping low-achieving peers improve was the best strategy to secure a bonus, leading many gifted students to mentor struggling peers. This approach resulted in significant and impressive improvements in learning during two implementations for first-year undergraduates.
Oliver (2006)	Australia	A public research university	Mixed method	12	Experiment designed by the authors, including discussions and observations	Both studies found that self-enhancement bias was linked to narcissism, ego involvement, self-serving attributions, and positive affect. Study 2 showed that self-enhancement correlated with declining self-esteem and well-being and increased disengagement from academics but did not predict higher performance or graduation rates.
Ostermaier (2018)	Germany	A public research university	Mixed method	1460	Quasi-experiment designed by authors	Early certificate awards and exam deadlines improved student performance, particularly among average students. These policies discouraged exam failures and retakes exam, highlighting the role of degree program structures in incentivizing academic success.
Palmiero et al. (2023)	Italy	A public research university	Mixed method	114	Trust scale, Guilford’s Alternative Uses, Generalized Anxiety Disorder 7 (GAD-7), Positive and Negative Affect Schedule (PANAS)	Divergent thinking quality (not quantity) positively predicted outgroup trust, while mood positively predicted ingroup trust. Divergent thinking task instructions had no effect on interpersonal trust. During the COVID-19 pandemic, higher-quality divergent thinking fostered outgroup trust, suggesting that individuals capable of generating uncommon and creative ideas are more inclusive and trusting of strangers.
Pandya (2023)	4 countries	Not specified	Mixed method	*n*_control_ = 56 *n*_experimental_ = 66	Short Form Self-Regulation Questionnaire (SSRQ), Emotion Regulation Questionnaire (ERQ), Scale of Positive and Negative Affect (SPANE), CAYCI Peer Relations Scale (CAYCI PRS), Ryff Psychological Well-being Scale (RPWBS)	Compared to an online affect management workshop, online spiritual lessons were more effective. Moderate effects were found on emotion regulation (cognitive reappraisal, expressive suppression) and well-being (autonomy, mastery, personal growth, relationships, purpose, and self-acceptance). High effects were observed on peer relations, self-regulation, affect balance, and overall well-being.
Parker et al. (2017)	US	A non-specified university	Mixed method	3908	Emotional Quotient Inventory (EQ-i: S)	High-achieving secondary students who completed an undergraduate degree scored significantly higher on several emotional intelligence (EI) dimensions than those who dropped out. Findings highlight the importance of EI in the successful transition to postsecondary education.
Patchan and Schunn (2016)	US	A public research university	Mixed method	189	Experiment designed by the authors	Lower-ability writers benefited more from feedback from lower-ability reviewers, while higher-ability writers benefited equally from both lower- and higher-ability reviewers.
Persson (2010)	Sweden	Mensa members	Survey	287	Questionnaire developed by the authors	The study found cause for concern, with primary school being a particularly hostile environment for gifted students. Conditions improved slightly in secondary and tertiary education, but dissatisfaction remained at all levels. Four key problem areas were identified: anti-intellectualism, unprepared teachers, lack of systemic support, and misdiagnosis by psychologists, leading to widespread dissatisfaction in an inclusive education system.
Peterson (2001)	US	Diverse institutions	Mixed method	4	Questionnaire developed by the authors, Experiment designed by the authors	Parent conflict was a dominant theme, with resolution linked to developmental progress, academic motivation, and emotional well-being. Differentiation involved identity exploration, career direction, and autonomy struggles during extended education.
Radulović et al. (2022)	Serbia	A public research university	Survey	83	Questionnaire developed by the authors, Didactic strategies and competencies of gifted students (DSCGS-1)	Students in Serbia had favorable views on gifted education and planned to incorporate positive concepts into teaching. They were also sensitive to students with disabilities, believing children with disabilities need special programs most. While satisfied with the teaching profession’s social status, they were less satisfied with its economic position. Overall, findings indicate a positive trend in support for gifted education in Serbia.
Ralston et al. (2017)	US	A non-specified university	Survey	40,143	Secondary data	Students’ challenges and needs vary based on their placement within an achievement quadrant. High-achievers required minimal intervention, while low-achievers needed comprehensive support. Overachievers mainly needed academic intervention, whereas underachievers, despite high potential, struggled with time management and self-discipline.
Rice et al. (2006)	US	A public university	Survey	499	Almost Perfect Scale (APS-R)	Adaptive and maladaptive perfectionism were significantly linked to stress, social connectedness, depression, hopelessness, and academic adjustment. However, some effects weakened when earlier distress and adjustment were controlled. Perceived stress and social connection moderated or mediated several effects.
Ridgley et al. (2022)	US	A non-specified university	Mixed method	126	Motivated Strategies for Learning Questionnaire (MSLQ), Quasi-experiment designed by authors	When solving difficult problems, students showed lower self-efficacy, performance evaluations, and effort, relying more on surface-level strategies instead of deeper approaches used for easier tasks. This suggests that gifted students may not transfer effective strategies to challenging tasks.
Riise et al. (2022)	Norway	Diverse institutions in Norway	Survey	14,000	Quasi experiment and secondary data analysis	Exposure to female general practitioners increased the likelihood of girls entering male-dominated STEMM fields in high school and college, especially for high-ability girls with low-educated mothers. This suggests that female role models enhance intergenerational mobility and reduce the gifted gap, even outside the classroom.
Rinn and Boazman (2014)	US	Two universities	Survey	357	Questionnaire developed by the authors Rotter’s Internal–External Locus of Control Scale (I-E Scale), Self-Description Questionnaire III (SDQ-III)	Locus of control did not significantly predict academic dishonesty for the non-honors group, though relationships were found among variables for the honors and aggregate groups. Additionally, academic self-concept was negatively related to academic dishonesty, indicating that students with lower academic self-concept were more likely to engage in dishonest behaviors.
Robins and Beer (2001)	US	A non-specified university	Mixed method	360	Questionnaire developed by the authors and data analysis, Positive and Negative Affect Schedule (PANAS), Self-evaluation, Narcissistic Personality Inventory (NPI)	Self-enhancement bias was linked to narcissism, ego involvement, self-serving attributions, and positive affect. Over time, self-enhancing students experienced declines in self-esteem, well-being, and academic engagement. However, self-enhancement did not predict higher academic performance or graduation rates, suggesting that while positive illusions may be beneficial in the short term, they are maladaptive in the long term.
Rodriguez-Nieto et al. (2019)	Mexico	A public university	Mixed method	74	Questionnaire developed by the authors	All groups reported high autotelic flow experiences, with psychomotor and imaginational overexcitability (OE) predicting 13% of the variance in global flow. The findings highlight the tensions and joy experienced by performing artists and athletes, emphasizing the need for educators, coaches, and psychologists to consider OEs, flow, and domain-specific differences in their support strategies.
Ruban and Reis (2006)	US	A research university	Mixed method	180	Motivated Strategies for Learning Questionnaire (MSLQ)	High-achievers used more advanced self-regulatory strategies like condensing notes, mnemonics, and concept mapping, while low-achievers relied more on surface-level strategies like flashcards and routine memorization.
Sa Ngiamsunthorn (2020)	Thailand	A public university	Mixed method	23	Questionnaire developed by the authors Observations	Challenge-based learning, problem-solving, project-based learning, well-designed questions, and in-depth learning styles effectively enhanced creative and insightful thinking. Using Facebook as an online learning platform further promoted discussion, collaboration, and critical thinking. Student feedback indicated that combining these methods created a more motivating and supportive learning environment, fostering creative thinking and satisfaction.
Salami et al. (2021)	US	A non-specified university	Survey	225	Student Adaptation to College Questionnaire (SACQ), Motivated Strategies for Learning Questionnaire (MSLQ), Multidimensional Perfectionism Scale (MPS), Perceived Stress Scale (PSS-10)	Racial microaggressions were significantly associated with increased worry about future employment among Black college students. However, perceived social support buffered this effect for low-achieving students but not for high-achieving students who remained vulnerable to potential isolation and academic pressure.
Saleh and Alali (2022)	Saudi Arabia	A public university	Survey	563	Questionnaire developed by the authors	Increased use of digital tools enhanced self-efficacy, leading to academic progress. Significant differences were observed in self-efficacy scores among gifted students.
Salem et al. (2022)	Egypt	Three universities	Survey	237	Scales for Rating the Behavioral Characteristics of Superior Students (SRBCSS), Generative Altruism Scale (GAIS), Cooperative/Competitive Strategy Scale (CCSS)	The study found a significant positive relationship between altruism and cooperation among gifted adolescents. Gender differences were observed in both traits, as well as differences between senior and junior students, favoring seniors.
Sbaih (2023)	Jordan	A public university	Survey	166	Torrance’s Tests of Creative Thinking (TTCT)	The study found higher Torrance Test of Creative Thinking scores with university level and slightly higher creativity in females. Creative thinking correlated positively with academic achievement, and originality (β = 0.40) was the strongest predictor, followed by fluency, flexibility, and elaboration.
Sedykh et al. (2021)	Russia	A public university	Mixed method	30	Questionnaire developed by the authors Observations	Developing leadership competencies in gifted students through a practice-oriented approach is widely recognized in education. Gamification enhances this process, making it more effective.
Shin et al. (2023)	South Korea	A public university	Survey	222	Ways of Coping Checklist (WCC), Korean Resilience Questionnaire (KRQ), Academic Self-Efficacy Scale (ASE), Big Five Inventory—Korean Version (BFI-K)	Academic high achievers had lower test anxiety, less neuroticism, higher self-efficacy, and less socially prescribed perfectionism than the comparison group. Neuroticism, test anxiety, and perfectionism were key predictors of academic performance. High achievers exhibited moderate test anxiety and perfectionism performing best on the College Scholastic Ability Test.
Siegle et al. (2010)	US	A public university	Survey	149	Questionnaire developed by the authors	The study found a positive relationship between students’ interest in a talent area and their self-assessed skill, strongest in non-academic areas. Their implicit theory of intelligence did not significantly affect their views on ability’s role in academic performance.
Sloan (2018)	US	Not applicable	Survey	1647	Archival data	The study found a significant difference (*p* < 0.05), with a higher percentage of NYC selective specialized high school graduates earning STEM degrees from an honors college compared to other high school graduates.
Smith (2006)	US	Not applicable	Survey	24,599	Archival data	High-achieving middle school students who experienced achievement loss during the transition to high school were more likely to leave their first college than those who did not experience achievement loss.
Snyder and Adelson (2017)	US	A public university	Survey	536	Questionnaire developed by the authors	The study developed and validated the Perceived Academic Underachievement Scale (PAUS) through two studies. Study 1 confirmed content validity and conducted an exploratory factor analysis, while Study 2 performed a confirmatory factor analysis and tested external validity. PAUS showed good internal consistency and loaded strongly onto a single factor, proving to be empirically distinct from related constructs.
Snyder et al. (2014)	US	A private university	Survey	108	Questionnaire developed by the authors	Students who received a fixed (entity) message about giftedness engaged in more behavioral self-handicapping after failure than those who received a growth (incremental) message. Among female participants, an entity message led to more claimed self-handicapping after failure and less after success, while an incremental message had no effect. Implicit messages did not influence male students claimed self-handicapping.
Tan et al. (2023)	Malaysia	A private university	Mixed method	10	Questionnaire developed by the authors and interviews	Gamification effectively increased underachievers’ learning interest. Key game elements, meaning, onboard tutorials, social pressure, and teams/guilds, were essential in motivating underachievers to participate in online lessons.
Thiemann (2022)	Switzerland	A public university	Survey	8073	Questionnaire	Low-ability students assigned to high-ability orientation week groups performed worse in their first year and had a higher dropout risk. Long-term effects included lower selection into popular majors (e.g., business administration) and lower final GPAs. Findings suggest that short-term peer group composition significantly impacts academic choices and long-term performance.
Thomson and Jaque (2016)	US	A public university	Survey	197	Overexcitabilities Questionnaire II (OEQII), Dispositional Flow Scale–2 (DFS-2).	Dancers and opera singers had higher overexcitability (OE) profiles than athletes. All groups reported high autotelic flow experiences, with psychomotor and imaginational OE predicting 13% of global flow variance. Findings highlight the tensions and joy linked to heightened OE in performing artists and athletes, emphasizing the need for educators, coaches, and psychologists to address domain-specific differences in training and support.
Tindage et al. (2024)	US	A public university	Survey	165	Questionnaire developed by the authors	Students of color (SOC) recalled both positive and negative memorable messages (MMs) about their academic ability. Positive MMs included praise, encouragement, advice, and support, while negative MMs involved criticism, discouragement, reprimand, and accusation. SOC often used positive MMs as microaffirmations to counter identity threats, while negative MMs were perceived as racial microaggressions, leading to self-doubt.
Tsai and Fu (2016)	Taiwan	A non-specified university	Mixed method	3	Student-problem score table, S-*p* chart, Questionnaire on Self-Concept and External Support System for Underachieving Gifted Students	Underachievement began in senior high school due to lack of motivation, reliance on memorization, counterproductive learning strategies, and low subject interest. Despite having clear career goals and positive self-concepts, students struggled with execution in professional development.
Tushnova (2020)	Russia	Not specified	Survey	76	Quasi-experiment designed by authors	The study found significant differences in self-assessed intelligence, social intelligence, and empathy. Mathematical generalization and practical mathematical thinking were strongly linked to social and perceptual abilities, including empathy, verbal expression recognition, and emotional management. Spatial reasoning skills correlated only with emotional intelligence.
Tyurikov et al. (2022)	Russia	Not specified	Survey	>6500	Questionnaire developed by the authors	Students pursuing further education highly valued research but lacked sufficient motivation tools. A healthy academic inbreeding model, based on open innovation, was proposed to retain talented graduates and enhance research engagement.
Van den Muijsenberg et al. (2021)	Belgium	Not specified	Mixed method	8	Questionnaire developed by the authors	Participants in the counseling program experienced behavioral changes, including improved study motivation, better time management, and enhanced study skills. Some students implemented study strategies more efficiently, while others realized the need for more intensive guidance, particularly for managing fear of failure. The long-term effects of the program showed sustained improvements in study habits and motivation.
Wai et al. (2005)	US	Not applicable	Survey	1975	Scholastic Assessment Test (SAT), Study of Values (SOV)	Early cognitive abilities (SAT scores) strongly correlated with later achievements, such as earning doctorates, higher income, obtaining patents, and securing tenure at top universities. Mathematical and verbal reasoning at age 13 effectively predicted future career paths and accomplishments, reinforcing the value of early talent identification and support.
Watters (2010)	Australia	Diverse institutions	Mixed method	200	Questionnaire developed by the authors, Interviews	Career decision-making among gifted students was influenced by both cognitive–personal variables and environmental factors. Teachers played a role in refining students’ career orientations rather than significantly changing them. Key teacher attributes that supported gifted students’ career interests included passion for the subject, strong content knowledge, making learning relevant, high expectations, clear explanations, and effective classroom management.
Woody and Parker (2012)	US	A non-specified university	Survey	86	Questionnaire developed by the authors	Participants relied on past musical experiences, sought peer support, and developed personalized strategies to succeed. Their self-identity as achievers played a key role, and many aimed to gain approval from friends and family to reconcile past experiences. The project re-engaged them with music-making and opened possibilities for future involvement.
Wu et al. (2022)	US	A public university	Survey	76	Questionnaire developed by the authors	Gifted students in an early college entrance program (NAASE) generally had positive perceptions of its effectiveness. Participants reported intellectual and social growth, strong peer and family relationships, and leadership development, though not all skills were equally impacted. Findings contribute to research on early college entrance effects on gifted students’ development and peer relationships.
Yusmansyah et al. (2019)	Indonesia	A public university	Mixed method	100	Questionnaire developed by the authors	Religious counseling services effectively enhanced mental strength in gifted students, helping them develop positive behaviors, essential for academic success. This support minimized internal obstacles, enabling students to complete their studies on time with satisfactory results.
Ziegler et al. (2019)	Different countries	Not applicable	Mixed method	529	Hamdan Matrices Test 4-6 (HMT 4-6), Teacher checklist 4-6 (HTC 4-6), Questionnaire of Educational and Learning Capital (QELC)	Educational and Learning Capital (ELC) predicted achievement beyond IQ in three domains: scholastics (sixth graders), STEM careers (women with STEM degrees), and athletics (long-distance runners). Successful individuals had greater access to and use of ELC. Findings support the Actiotope Model of Giftedness, highlighting that talent development depends on person–environment interactions, with social–emotional support and high-quality learning resources playing key roles in success.

## Data Availability

The data presented in this article is available on requests with justification and should be directed to marjolijn.vanweerdenburg@ru.nl.

## Appendix A

### Social–Emotional Needs per Category

**Social–Emotional Needs**	**Hits**
Category 1: Self-Perception, Motivation, and Performance Expectations	
Motivation and self-regulation	63
Self-perception, confidence, self-efficacy	61
Attitudes toward school and teachers	52
Desire for autonomy/independence learning	39
Coping with performance pressure	34
Need for adequate expectations and self-appraisal	17
Category 2: Social Integration and Acceptance	
Sense of belonging and social acceptance	45
Social interactions, friendships, and relations	35
Trust and emotional validation from others	32
Parental and family expectations	20
Social perceptions and misalignment	19
Leadership and social responsibility	17
Category 3: Psychological Well-Being and Emotional Regulation	
Need for support and understanding	48
Sense of control over success and failure	46
Anxiety and stress management	36
Identity formation and career choices	33
Emotional intelligence and regulation	18
Spiritual and existential well-being	10
Emotional overexcitability and sensitivity	10
Need for stability	6

## Appendix B

### Educational Needs per Category

**Educational Needs**	**Hits**
Category 1: Curriculum Design and Instructional Strategies	
Personalized and differentiated learning	54
Instructional strategies and (dynamic or formative) assessment	50
Academic challenge and autonomy	50
Higher-order thinking, critical thinking, and problem-solving	32
Curiosity, active and inquiry-based learning	25
Collaborative and mastery-based learning	22
Learning agility/study skills	16
Language and literacy development	10
STEM and research-based education	10
Technology-enhanced learning	10
Real-world and applied learning	10
Category 2: Academic and Social Support Systems	
Academic support and flexibility	48
Mentorship and career guidance	41
Counseling and mental health support	35
Research and experiential learning opportunities	28
University transition and social integration	26
Peer-to-peer learning assistance/feedback	15
Mastery experiences	9
University campus experiences	9
Financial and accessibility support	6
Category 3: Teacher Training and Pedagogical Expertise	
Instructional approaches for gifted learners	51
Student support and well-being (by teachers)	44
Engagement and inclusion strategies	36
Professional development teachers	20
Early and targeted interventions	20
Positive scientific attitude from teachers	14
